# Middle Eastern nurses’ views/experiences of work and well-being with the support measures during past disease outbreaks and COVID-19: a qualitative systematic review

**DOI:** 10.1186/s12912-023-01343-4

**Published:** 2023-07-03

**Authors:** Sara Ahmed Marair, Nigel Slater

**Affiliations:** 1 King Saud Medical City, Riyadh, Saudi Arabia; 2 University of Nottingham, Nottingham, NG1 5NT UK

**Keywords:** Nursing staff, Hospital, Well-being, Middle Eastern nurses, Support measures, Past disease outbreaks, COVID-19, Psychological distress, Professional burnout, Psychological intervention, Health care organisations

## Abstract

**Background:**

The potential psychological health impact of pandemics on nurses has been increasingly widely recognised, as have recommendations to establish support measures for nurses’ well-being. Despite the availability of support measures significant number of nurses still experienced burnout and mental distress during Covid-19. Few efforts have been made in the wider literature to understand how nurses experience well-being support or how they perceive it affects their well-being during pandemics. In the Middle East, understanding and exploring well-being support measures during pandemics from nurses’ perspectives has not received significant attention.

**Objective:**

To investigate nurses’ perspectives and experiences of well-being support measures during prior pandemics and the Covid-19 pandemic in the Middle East.

**Methods:**

A systematic qualitative review was undertaken utilising the JBI model as a framework. Searches were carried out in databases comprised CINAHL, MEDLINE, NUsearch Library of Nottingham University and Google Scholar. Moreover, a manual search through reference lists for relevant studies were carried out.

**Data extraction and synthesis:**

Eleven studies were included in the review. The findings from the included qualitative studies were extracted using the JBI-QARI data extraction tool for qualitative research. The results were synthesised using a meta-synthesis in line with the JBI approach.

**Results:**

The included studies yielded an aggregate of 111 findings and were categorised into 14 categories, followed by four synthesised findings. These were: [1] nurses experienced challenges during MERS, yet different strategies were implemented by leaders and nurses to manage these challenges; [2] some well-being support measures were unfulfilled during Covid-19; [3] additional aspects compounded negatively on nurses’ well- being; and [4] nurses showed maturity during Covid-19.

**Conclusion:**

In comparison to prior health emergencies, well-being support measures during Covid-19 were not sufficiently adopted. Nurse policymakers and managers should consider these support measures to correspond with nurses’ needs and explore the contextual factors that affect their implementation.

**Trial registration:**

PROSPERO (CRD42022344005).

**Supplementary Information:**

The online version contains supplementary material available at 10.1186/s12912-023-01343-4.

## Introduction

There have been several disease outbreaks throughout the past years. Notably, pandemics such as severe acute respiratory syndrome (SARS), Middle East respiratory syndrome (MERS), and the H1N1 influenza have revealed how healthcare systems dealt with massive morbidity and fatality rates in the past two decades [1, 2]. Likewise, when the global Covid-19 pandemic was declared by the World Health Organisation (WHO) [3] countries around the world were urged to take immediate action.

Coronavirus is partially defined as the occurrence of respiratory disease and can be transmitted in a variety of ways, including by direct transmission, contact transmission, and airborne transmissions [4]. Consequently, it has infected many healthcare professionals and has impacted the entire world significantly [5, 6]. Indeed, according to Halcomb [7] the current pandemic of Covid-19 presents a global health emergency of a level never seen in our lifetimes.

Accordingly, healthcare system responses to the pandemic and the ability of acute care facilities to fulfil the needs of infected patients with Covid-19 have received huge global attention [7], with a major focus on healthcare professionals in critical and acute care settings, primarily nurses. Healthcare systems worldwide rely heavily on nurses, which has become more evident than ever during Covid-19. Meanwhile, Jackson [8] affirms that owing to widespread disease outbreaks, there is now a higher demand than there has ever been before for nurses. However, as mentioned previously, this global epidemic has infected tremendous numbers of nurses leading to a substantial increase in the fatality rate [9].

For instance, Italy identified that a significant percentage of the total infections were in healthcare professionals [10]. Additionally, an editorial by The Lancet [11] report that over 3000 healthcare professionals in China were infected at the onset of the pandemic, with twenty-two dying.

Therefore, it is important to highlight how nurses are particularly vulnerable to communicable disease infections that can be spread through contact with blood and body fluids and exposure to airborne microbes [12]. Essentially, this results from the nature of their work. For example, they are exposed to communicable diseases more frequently owing to their direct patient care activities [13]. Equally, nurses are required as part of their roles to demonstrate genuine care and empathy for patients’ needs, which requires a high level of emotional involvement on their part [14].

Certainly, providing high-quality care while protecting themselves from a disease epidemic may be challenging for nurses. This can burden them, and place further strain their already-overburdened workloads during Covid-19. Therefore, the overwhelming experience of such a disease outbreak could have both short- and long-term effects on the psychological health of nurses without adequate support and appropriate preparation [15].

Unsurprisingly, therefore, nurses have reported increased rates of mental and emotional distress throughout pandemics, thereby impacting their ability to maintain their wellbeing. It was widely reported in 2003 that SARS had a significant psychological impact on healthcare workers (HCWs). For example, significant emotional distress was found in up to 57% of HCWs and was linked to quarantine, fear of contamination, worries about family, work stress, and social stigma [16].

Notably, this is similar to what was experienced during the MERS outbreaks, with healthcare providers' stress and anxieties at work having negative impacts on their overall efficacy [17]. These detrimental experiences were also evident during Covid-19, with Shahrour and Dardas [18] stating that during the Covid-19 pandemic HCWs – but primarily nurses were more vulnerable to experiencing post-traumatic stress disorder and high stress levels. Such similarity around the impact of disease outbreaks on nurses’ experiences is not surprising. Indeed, Van Mol [19] highlighted that working in a very stressful environment can have a detrimental effect on a medical professional's overall health and wellbeing.

## Background

### What do we already know about the topic?

According to prior disease outbreaks' adverse effects on the wellbeing of nurses, many exhibited various levels of distress, anxiety, and fears depending on the contributing factors they encountered. For instance, Goulia [13] described how one of the most common worries reported by nurses during the H1N1 outbreak was that the disease could be transmitted to their loved ones and negatively affect their wellbeing.

Other studies found that distress can be exacerbated by a lack of reliable information that healthcare organisations should ideally provide in the early stages of a disease outbreak [20]. Moreover, some believe that fear of stigmatisation in the form of avoidance by relatives and friends was also identified as a significant aspect of many healthcare professionals' experience during the SARS outbreak [21].

In contrast, Kim and Choi [22] assert that the shortage of hospital resources such as personal protective equipment (PPE) was the most significant determinant in MERS related burnout, followed by insufficient support from family members and friends. Furthermore, an overburdened healthcare system has been associated with nurses’ mental distress in earlier outbreaks of infectious diseases, alongside the physical and emotional strain [2]. Ultimately, the pandemics profoundly impacted the wellbeing of nurses in different aspects of their work.

Despite the devastating experience of nurses in past disease outbreaks, well-being supportive measures have nonetheless been recommended to preserve the wellbeing of nurses. The term well-being support measures emphasises the significance of advocating for healthcare workers' mental health and well-being using a variety of measures [23]. Specifically, providing training and counselling to nursing staff, alongside timely and reliable information at the onset of any global pandemic, could be regarded as the most effective strategies to mitigate the psychological impact of the crisis on nurses [24]. Other findings demonstrate that the psychological health of nurses caring for patients with communicable diseases might be significantly improved by executing an intervention programme that incorporates all dimensions of stigma, resilience and stress [25]. Crucially, it was also suggested that preparations should be considered to avoid burnout and to manage and resolve work-related stress, in addition to the availability of hospital supply and strengthening the support of family and friends [22]. Evidently, numerous supportive measures were advocated in prior disease outbreaks to mitigate’any negative impacts on nurses.

### Middle east context

**Fig. 1:**
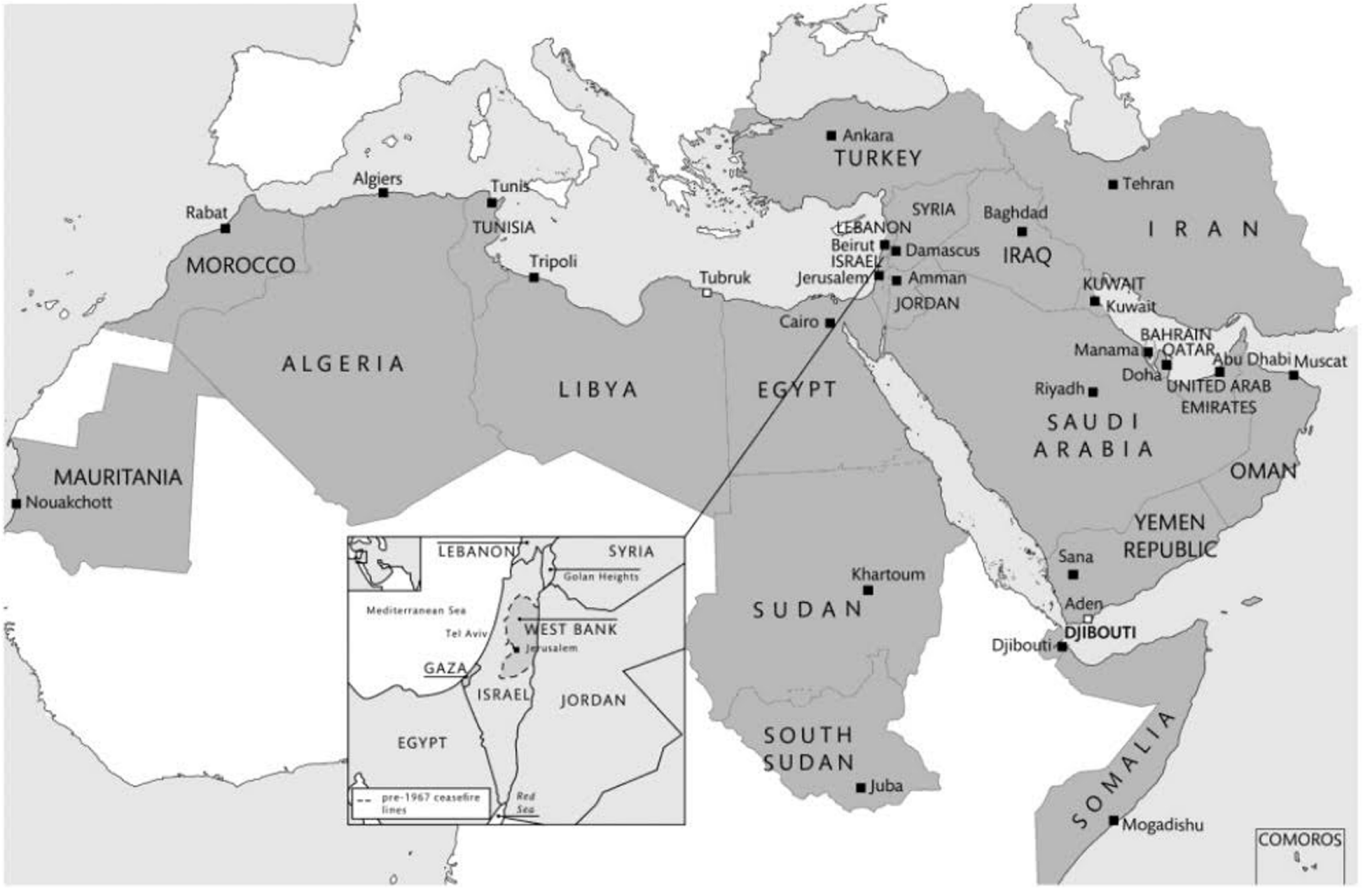
Map of the Middle East

The Middle East refers to the Arabian Peninsula states; non-Arab countries such as Iran, Israel and Turkey are included, with West Asia and North Africa's Arab League seen in Fig. 1 [26]. The Middle East, North Africa, and parts of Asia and Africa where populations are mostly Muslim are frequently referred to as the ‘Arab world’ or the ‘Muslim world’ [27].

Fawcett L. International relations of the Middle East. 4th ed. Oxford University Press; 2016.

The Covid-19 pandemic presents a significant worldwide threat to under- resourced healthcare services [28]. Similar to the Middle East, the International Health Regulations for Middle Eastern countries necessitate the preparation of action plans for worldwide public health emergencies [29].

However, the Covid-19 pandemic may pose considerable obstacles to certain countries in the Middle East with underdeveloped or inadequate healthcare systems [30]. Consequently, Egypt escalated its protective measures, including a partial lockdown, with nurses likely to be a key link in the spread of Covid-19 owing to their frequent interaction with infected patients [28].

Furthermore, Iran was ranked sixth in highest incidences of Covid-19 mortality next to Italy and China. Indeed, despite reports of the outbreak being publicised regularly within Iran's hospitals, the health service’s preparation for the pandemic were not particularly successful [31]. Equally, however, a study in Turkey reported that the Covid-19 pandemic remains a challenge that even the most advanced systems of the modern age have failed to tackle [32]. It is important to note that the Covid-19 crisis is more than just a health service issue—it significantly impacts entire populations’ and HCWs' quality of life, in addition to their social interactions [33]. Another critical point to acknowledge is that there may be underlying religious or spiritual influences on this region's healthcare systems and populations owing to the large Muslim populations [29].

Certainly, previous pandemic experiences demonstrate that they pose significant threats to the wellbeing of nurses. Unfortunately, there is a dearth of evidence on the effects of the Covid-19 pandemic on nurses' mental health, particularly in the Middle Eastern region [34].

For example, a study in Egypt by El-Monshed [28] highlighted that limited studies have been undertaken on Covid-19 and nursing, specifically, nurses' understanding, worries, perception of impact, and preparedness for Covid-19 are unknown.

Similarly, in Saudi Arabia, Al Ateeq [35] stressed that there are very few relevant studies that have examined the possibility of frontline nurses being at greater risk of poor mental health outcomes. Likewise, a study in Iran emphasised that while research has focused on Covid-19 patients, few have truly assessed the issues that ICU nurses confront when caring for these patients [31]. Interestingly, this lack of evidence was not only witnessed during Covid-19 but also during nurses’ experiences with the MERS outbreak. This included their feelings, concerns, and coping mechanisms having not been explored effectively [36]. Considering these issues, it was necessary to undertake a systematic qualitative review that explored nurses' perspectives on and experiences with previously proposed supporting measures in the Middle East, rather than limiting the scope to nurses' experience with pandemics.

### The state of nursing well-being (relevance to advanced nursing)

The rationale of this systematic review is illustrated by the fact that despite earlier studies advocating different support measures to maintain nursing well-being during future pandemic, Covid-19 pandemic has had a major adverse effects on nurse well-being. Understanding that there are still several unknowns concerning the current scenario is vital. Accordingly, it is essential to highlight that advanced nursing practice is the aspect of nursing that is best prepared to meet today's demands and difficulties. This is particularly relevant to the Covid-19 pandemic's quickly evolving healthcare environment [37].

Advanced nursing roles are formed by integrating clinical practice with education, leadership, professional development, evidence-based practice and research [38]. One study reported that the psychosocial aspect of care has both a personal and a professional impact on nurses [39].

Thus, by preserving their physical and mental health, nurses strive to advance their practice, as the benefits of high well-being include thriving in the profession, employee engagement and organisational commitment [40]. On that account, a positive well-being experience can enhance practice growth and thus contribute to clinical practice, one of the four pillars of advanced nursing practice [41].

Furthermore, patients, families and the healthcare system are all influenced by advanced nursing practice's role in clinical practice, including clinical decision making, knowledge, education and caring practice [42]. Addressing nurses' inability to obtain or use information in practice promotes the quality of patient care, nurses' experiences and nurse retention rates [37]. Additionally, there is a direct and crucial link between nursing clinical practice and the work environment with patient satisfaction and outcomes [39]. Therefore, by maintaining their well-being, nurses strengthen their practice to manage and respond to changing and challenging situations, such as the Covid-19 pandemic.

Leadership is another essential element of the four pillars that supports advanced nursing [41]. Management and leadership in advanced nursing include determining the need for development and improvements in clinical practice, setting clear goals and bringing together an effective team to accomplish change [41]. It is playing a major role as well in creating employee experiences, which significantly influence nurses' worke environment [40].

Despite that, there is a dearth of evidence regarding the effects of Covid- 19 and previous pandemics on the psychological health of nurses, particularly in the Middle East [34, 36]. The necessity for leaders to recognise the importance of addressing these research gaps and areas in which information is limited may help promote nurse well-being, maintain patient safety and promote quality of care in the Middle East.

However, since leadership is one of the pillars of advanced nursing and correlated with employee behaviour, performance and well-being [43]. Leaders must have a comprehensive understanding of the support measures that preserve nurses' well-being so that they can continue to offer quality care to patients throughout the Covid-19 pandemic and in future [7]. On that account, this review is significant to advanced nursing.

### Aim

This review aims to synthesise and provide evidence in a systematic review; explore nurses' perceptions of and experiences with well-being supports measures during the Covid-19 pandemic; and compare and contrast these experiences with those during past disease outbreaks in the Middle East, such as influenza, SARS and MERS. This review is not limited to nurses' experiences and perspectives with pandemics, but focuses on their views and experiences with previously recommended support measures during Covid-19 in the Middle East. It is also investigating if the support measures were altered during the Covid-19 pandemic and how this affected nurse well-being.

### Objectives

1. Exploring nurses' perspectives and experiences with support measures during previous pandemics and Covid-19.
2. Examining which of the support measures have been altered during Covid-19.
3. Understanding if nurses faced unique challenges during Covid-19 to maintain their well-being.
4. Surveying what might be different during Covid-19 regarding their well-being and whether their cultural and religious beliefs had any influence on it.

### Justification for the review question

Several significant aspects are relevant to conducting a systematic qualitative review. To begin with, there is a paucity of evidence regarding well-being support measures and their effects on nurses during the Covid- 19 pandemic, particularly in the Middle East. This lack of evidence is not unique to Covid-19 and also applies to nurses' experiences with past disease outbreaks in the Middle East, which have not been adequately investigated. Another prominent point to consider is that prior to initiating this systematic review, a search of the PROSPERO, MEDLINE and CINAHL databases yielded no previously conducted qualitative systematic reviews that investigate the influence of support measures on nurses’ well- being during pandemics in the Middle East. There is an unpublished dissertation by AlRehaili in the Middle East that only investigates nurses’ experience with Covid-19, but it does not go beyond the scope of the present review. However, cultural and religious norms may influence these factors, or they may require accompaniment by additional factors to sustain favourable nurse experiences.

Considering these findings, it is relevant to conduct a qualitative systematic review that is not limited to nurses' experiences and perspectives with pandemics, but also focus on their views and experiences with previous support measures in the Middle East during Covid-19 in comparison to past pandemics. This will provide a broader and deeper understanding of issues related to well-being in this region. Ultimately, the main question that this systematic review investigates is:

Have the well-being support measures that were implemented in previous pandemics influenced nurses’ well-being during the Covid-19 pandemic in the Middle East?

## Method

### The approach adopted for systematic reviews

This review aims to better understand nurses' experiences and views with well-being support measures during the Covid-19 pandemic and prior pandemics in the Middle East and to get a clear insight into how these measures impacted nurses’ well-being during Covid-19.

Therefore, it was important to conduct a systematic qualitative study that would provide a profound and broad knowledge of the support measures associated with well-being in the Middle East rather than limiting the study’s scope to nurses’ viewpoints and experiences of pandemics.

Moreover, the question was a qualitative question, which required a qualitative systematic review. Because of the need to capture and understand nurses’ perceptions and experiences with these measures, the JBI meta-aggregation approach was adapted for this systematic review. Meta-aggregation is also the recommended JBI method for answering qualitative questions and generating further recommendations [44].

This review was registered in PROSPERO (CRD42022344005).

### Systematic review method

When conducting a systematic review, it is crucial to adhere to a specific method to attain the essential rigorous standards and eliminate the possibility of bias in the review steps. Therefore, it is essential to review the objectives, questions, inclusion and exclusion criteria, search strategies, critical appraisals, data extractions, data syntheses, and confidence assessments of methods [45]. Clear reporting of the methods applied for the synthesis is required, and this is a defining characteristic of this review.

### Review question

Review questions provide a framework for creating a comprehensive review report by guiding and directing the formulation of review criteria and facilitating more efficient searching [46]. Accordingly, the format for a well-defined qualitative research question – population, the phenomenon of interest and context (PICo) – is presented in Table 1below. It was adopted from a JBI systematic review [44].

**Table 1:** PICo framework

PICo	Application to research questions
**P-** Population	Nurses in in-patient settings during Covid-19 and past disease outbreaks such as severe acute respiratory syndrome (SARS), Middle East respiratory syndrome (MERS), and H1N1 influenza
**I**- phenomenon of interest	Nurses' perspectives and experiences with support measures during the Covid-19 and past disease outbreaks
**Co**- context	Hospitals in Middle East region

Ultimately, the main question that this systematic review investigates is:

Have the well-being support measures that were implemented in previous pandemics influenced nurses’ well-being during the Covid-19 pandemic in the Middle East?

### Inclusion and exclusion criteria according to PICo

Establishing inclusion and exclusion criteria minimises the possibility of bias and increases a review’s reliability [46]. The inclusion criteria presented in Table 1 were applied to this review and the exclusion criteria are further explained in the characteristics of the included and exclusion studies in Table 2.

**Table 2:** Characteristics of the inclusion and exclusion criteria of primary studies

Inclusion criteria	Exclusion criteria
Qualitative studies	Not qualitative studies
Studies explore nurses' experiences with the support measures during covid-19 or with past disease outbreaks (H1N1, MERS, SARS)	Studies exploring the experiences of nurses with other crisis such as natural crisis or wars
Studies focus on nurses' experiences during the Covid-19 pandemic and previous disease outbreaks	Studies exploring the experiences of student nurses’ or community nurses
studies were conducted in the Middle East	Studies not in the Middle East
In any area in the hospital setting	Out of the hospital settings (primary care and community settings)
Studies published in English	Studies published in other languages
studies involving nurses as part of a representative sample of health care providers	Studies investigate the experience of health care providers without including nurses
Studies were conducted from January 2003 onwards	-
Peer reviewed studies	Non peer reviewed

### Type of study

The types of studies selected for a systematic review play a significant role in determining the validity of the study design and the reliability of the results [47]. Therefore, this review included empirical qualitative studies that explored nurses’ experiences of supported measures or focused on nurses’ experiences during the Covid- 19 pandemic and previous pandemics in the Middle East. However, due to the timing of the master’s dissertation framework, only published peer- reviewed studies and studies published in the English language were included. Additionally, data from unpublished or non-peer-reviewed studies can alter a systematic review’s results [45]. The inclusion and exclusion criteria for the studies are summarised in Table 2 below.

### Search strategy

To compile a comprehensive list of possibly relevant studies, it was necessary to undertake a thorough literature search [48]. This began with a primary search of major databases, such as MEDLINE, using keywords identified from the review question. This preliminary search identified the most relevant search phrases, such as additional keywords, subject headings, and indexing terms, which were then used to search all the included databases, such as CINAHL (EBSCO) and MEDLINE (Ovid). In addition, further searches were conducted using Google Scholar and Nottingham University’s NUsearch library. This was followed by manually searching the reference lists of the tracked relevant studies. To ensure that only the most current past pandemics, such as SARS, H1N1 influenza, MERS and Covid-19 were included, searches were limited to 2003 onward.

**Fig. 2:**
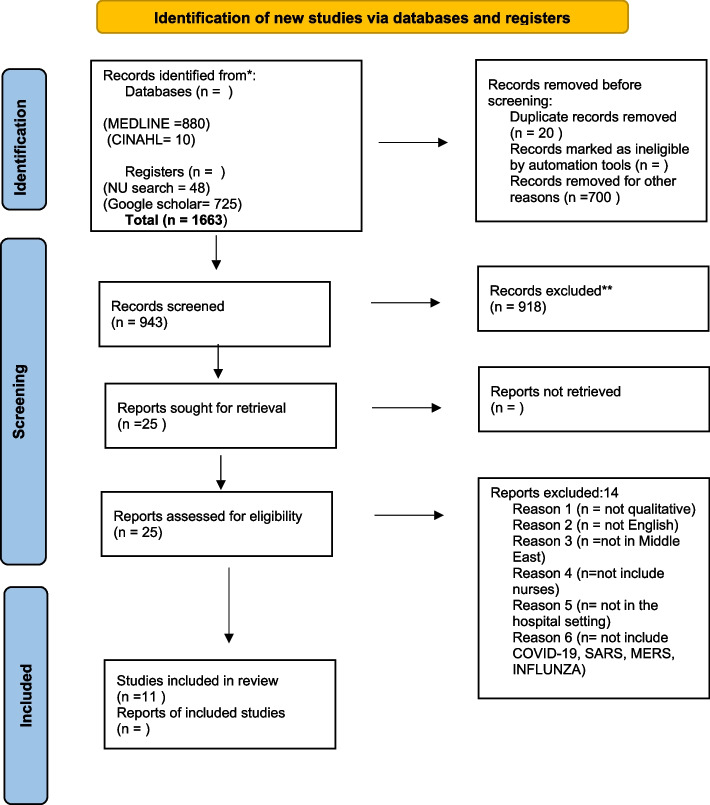
PRISMA flow diagram. Page M, McKenzie J, Bossuyt P, Boutron I, Hoffmann T, Mulrow C, and Moher D. The PRISMA 2020 statement: an updated guideline for reporting systematic reviews. BMJ.2021;372 (71). http://www.prisma-statement.org/. Accessed 15 November 2021

The database search strategy and the search phrases are presented in detail in Appendix 1 (see Additional file 1). Additionally, the search results and the study’s inclusion and exclusion criteria were mapped out using an updated Preferred Reporting Items for Systematic Reviews and Meta-Analyses (PRISMA) flowchart (Fig. 2).

### Assessment of the quality of the methodology

Before including a study in the review, two reviewers examined all the search results, including the titles and abstracts, and excluded any articles that did not fit the inclusion criteria. Retrieved full-text articles were assessed again considering the inclusion and exclusion criteria.

It is recommended that at least two reviewers carry out the initial screening of titles and abstracts according to the research question, study design and population [45], therefore this process conducted independently by two reviewers.

Since a critical appraisal is an essential element of conducting a systematic review [49], two reviewers examined each paper’s methodological validity. The JBI’s tool, used to perform the systematic appraisal of the qualitative studies [44], was selected because it has been found to be the most coherent tool in contrast to other appraisal methods for qualitative research [50]. Each study in the review was evaluated by two reviewers who agreed using a set of 10 questions from the qualitative appraisal tool to rate the quality of each study. The appraisal questions could only be answered by "yes", "no", or "unclear", and each "yes" response received a score of 1, each "No" response received a score of 0, and each "unclear" response received a score of 0.5 (see Appendix 2 in Additional file 1). The quality of the qualitative studies was rated on a scale from 0 to 10, with higher scores indicating higher overall quality.

### Data extraction and synthesis

A significant aspect of a systematic review, which is distinct from traditional literature reviews, is the process of extracting, synthesising, and merging data from multiple research studies [46]. Thus, the results from the included qualitative studies were extracted by two reviewers using the JBI- QARI data extraction tool for qualitative research [44]. The extracted data included specific information regarding the methodology, method, phenomena of interest, setting, geographical contexts, participants, method of data analysis and author’s conclusion (see Appendix 3 in Additional file 1).

This review used a meta-aggregative strategy to synthesise the results obtained from the included studies (see Appendix 4 in Additional file 1). The collected findings were categorised to generate thoroughly synthesised results that reflected the aggregation and to provide solid basis evidence for EBP [44].

### Confidence assessment of the review findings

This review considered the dependability and credibility of the included studies to determine the confidence of the synthesised qualitative findings. As a result, a table of findings summaries was provided by applying ConQual, representing an overall ranking of the confidence assessment of the review findings (see Appendix 5 in Additional file 1). In ConQual, the dependability rating is determined by particular questions from the critical appraisal tool, and the credibility is determined by assessing the level of the credibility of the synthesised finding [44].

It is worth highlighting that Munn [46] state that the reviewers of qualitative systematic reviews benefit from using the ConQual approach because it can give them confidence in the evidence and aid decision-making.

## Result

### Results of literature search

This literature search was carried out on 30 November 2021 and overall, 1663 studies were identified. A total of 10 studies from CINAHL (EBSCO), 880 from MEDLINE (Ovid), 48 from the NUsearch library of Nottingham University, and 725 from Google Scholar were found.

Of 1663 identified studies, 20 were removed as duplicates and 700 records were screened by title to exclude irrelevant research. This resulted in 943 records remaining. Following abstract screening to verify that the inclusion criteria for this systematic review were met, 918 articles were discarded. This then left 25 articles to be read the full text, but out of these 14 studies were excluded as they did not meet the inclusion criteria of this review (Appendix 6 see Additional file 1). Among the remaining articles, 9 eligible studies were identified through the electronic literature search. Additionally, by manual search in the reference list of the eligible studies, 2 additional studies were identified, bringing the total number of the included studies that met the inclusion criteria to 11.

Importantly, despite a thorough search of the available databases for relevant literature and the assistance of the librarian research team at Nottingham University, no qualitative studies were found that involved nurses and had been conducted in the Middle East during previous pandemics. Only two studies were identified via a manual search as mentioned above.

The process used to identify the search result sample is displayed (an updated PRISMA chart) in Fig. 2. Further, the search terms used during literature searches are presented in Appendix 1 (see Additional file 1).

### Methodological quality of the studies

The JBI appraisal tool was utilised to appraise 11 studies that were ultimately included in the review (see Appendix 2 in Additional file 1). Each study in the review was evaluated using a set of 10 questions from the JBI appraisal tool for qualitative studies to rate the quality of each study. These questions are determined by ‘yes’ or ‘no’ or ‘unclear’; however, each question was allocated a score (yes = 1, No = 0 and unclear = 0.5), with the total score indicating overall quality.

Five studies were funded [51–55] this might lead to bias in the results or during the process of conducting the research. According to Cohen [56] some studies show bias in drawing conclusions that are favourable to the sponsor's interest. Further, in one study the researcher's statement on their influence over the research results was unclear [57] in another article, the author acknowledged knowing six participants; however, no methods were identified to address the potential for interviewer bias as a result [58]. Additionally, in one study, the statement locating the researcher culturally or theoretically was unclear [55].

Moreover, in a study by Kalateh-Sadati [59] the conclusions drawn in the research were also unclear; the interview was conducted in the Persian language, but the study questions and participant voices were presented in the English language which is relevant to my inclusion criteria. Another issue in this study is that the participants' names were mentioned and it was not clearly stated if these were pseudonyms or their actual names. Overall, none of these studies was excluded due to the limited number of studies conducted in the Middle East.

### Characteristics of the included studies

All 11 studies included in this review were published from 2003 onward. All the identified studies were published in English, carried out during prior and current disease outbreaks. However, only two studies covered one past pandemic MERS [60, 55] and the remaining studies were conducted during Covid-19. All the included studies conducted in the Middle East (albeit only in four countries). Five studies were carried out in Iran [59, 52, 51, 54, 57]; two studies were conducted in Saudi Arabia [60, 55]; one study was conducted in Qatar [53], and three were carried out in Turkey [61, 62, 58].

The eleven studies all employed different qualitative methodologies. A qualitative conventional content analysis was utilised in one study [57]. Three of the research studies only mentioned 'qualitative study' [59, 54, 55]. The phenomenology approach was employed in five studies [52, 58, 62, 53, 61]. One applied a qualitative descriptive approac [51]. Another one applied qualitative methods of process evaluation [60].

All included studies employed interviews, though some had to be conducted slightly differently due to safety precautions during disease outbreaks. For instance, one study employed a mixed interview, and it was conducted either face to face or over the telephone [52]. Four studies employed face-to-face interviews [51, 53, 57, 55].

Three used online interviews [58, 62, 61]. One study used telephone interviews [54]. Another study used individual and group interviews [60]. However, in one study the method used to interview participants was not described [59].

A total of 169 nurses working in hospitals participated in the 11 studies. However, since two studies [60, 59] did not specify the gender breakdown of the participants, it was difficult to determine the proportion of female to male nurses.

Nevertheless, since the remaining studies provided detailed gender breakdowns for the participant, it seems the ratio of female to male nurses skews towards female nurses. Moreover, all studies that were included had their research approved by an ethics committee and had informed consent from all participants. Appendix 7 in Additional file 1 presents additional information regarding the included studies.

### Data extraction and meta-synthesis

A total of 111 findings were extracted from the included studies. The findings from each study were extracted from the underlying themes and subcategories. Additionally, in the studies that only offered their results as themes without subthemes, the findings were identified through repeated readings of the text to extract the themes as a finding.

All the extracted findings were supported with direct quotes from the participants listed in Appendix 8 in the Additional file 1. In addition, the findings' credibility was assessed; 98 were unequivocal, 13 were credible, and none were unsupported.

Using meta-aggregation principles, findings were categorised based on their similar meaning and concepts and then synthesised. It is noteworthy that the studies from prior epidemics and Covid-19 were aggregated separately. Overall, 14 categories were developed from the findings and four synthesised findings will be discussed below.

### Four categories from past pandemics

1. Nurses experienced organisational obstacles.
2. Nurses experienced different social challenges.
3. The negative emotions and thoughts related to their fear of being infected and severity of disease.
4. Factors aid in managing the pandemic challenges at organisation level and the individual level.

### Ten categories from Covid-19 pandemic

1. Organisations' inefficiency in supporting nurses with adequate resources, preparedness and information about the disease.
2. Psychological distress associated with challenging hospital environments and caring for Covid-19 patients.
3. The experience of social support varied amongst nurses.
4. Experiences around PPE.
5. The fear of contracting an infection affected nurses' psychological well-being and their life outside of their clinical role.
6. The psychological needs and concerns of nurses were not addressed.
7. Professional turnover intention.
8. Different psychosocial and behavioural resilience approaches were adopted by nurses.
9. Commitment to the nursing profession.
10. Advancing in nursing practice.

### Meta-synthesis one: nurses experienced challenges during MERS, yet different strategies were implemented by leaders and nurses to manage these challenges

Four categories contributed from this meta-synthesis: [1] nurses experienced organisational obstacles, [2] nurses experienced different social challenges, [3] the negative emotions and thoughts related to their fear of being infected and severity of disease and [4] factors aid in managing the pandemic challenges at organisation level and the individual level.

Findings in category one reveal that nurses viewed the lack of preparedness of healthcare organisations in many aspects as contributing to the challenges they faced throughout the outbreak. Some described how the infection control guidelines were not adequately implemented, as alluded to in this statement, "Create a policy where you alert the staff as soon as you have one case or two cases in the ER; you [i.e. the decision makers] should have alerted all the staff" [55] ^(p190)^. While others considered poor communication from leaders and inadequate information about disease created additional obstacles [60].

The second category described nurses' negative experiences within the community, which negatively impacted their emotional well-being. These included behaviours such as social isolation and rejection as one nurse commented, "I felt bad... it feels like I'm the dirtiest person in the world; that's why they have to avoid me. I can't approach them because they are terrified, you know. I felt bad" [55] ^(p189)^. In addition, the experience of stigmatisation by nurses was also experienced when interacting with public [55].

Findings from category three illustrate that nurses were anxious about contracting the disease and its potentially devastating effects on their well- being. It was described in this statement by one nurse, "So, even I have this kind of thinking, Oh my God, after few days I will die, after few days I will get intubated, or something like this like that I was really scared if I will survive or if I will be gone that time" [55] ^(p189)^ . Another aspect that has been recognised is that the medical knowledge of nurses exacerbated their worries by adding additional stress, such as picturing inevitable events that could occur [55].

Despite the above reported challenges, findings from category four identified support measures used to overcome the negative experience. For example, at organisation level, building trust and teamwork, effective leadership style and providing enough information regarding the Infection control measures [60]. As asserted by one nurse, "We– we had a lot of support from Infection control department…lots of information available. Um, the Infection control practitioners were on the units day and night, supporting the staff. Management as well….in terms of supplies, equipment, all of that, we did not have any issues. It was supplied – readily available" [60] ^(p 6)^ . At an individual level, nurses adopted resilience behaviours to cope with the pandemic; for instance, they viewed their survival as a reward from God for caring for their patients [55].

### Meta-synthesis two: some well-being support measures were unfulfilled during COVID-19

This meta-synthesis consisted of four categories [1] organisations' inefficiency in supporting nurses with adequate resources, preparedness and information about the disease, [2] psychological distress associated with challenging hospital environments and caring for Covid-19 patients, [3] the experience of social support varied amongst nurses and [4] experiences around PPE.

Findings from category one identified a variety of perspectives regarding the reasons for the limited support nurses perceived from their organisation. The participants reported that a lack of staff and resources within their organisation had a detrimental effect on their mental well-being [59, 58]. Another issue highlighted is the lack of visibility of leaders, as expressed in the statement, "We expect officials to come and visit us, motivate us, and boost our morale. Since the outbreak of Coronavirus, no university deputies or hospital managers have come to ask ‘What are you doing here? What kinds of problems are you facing?’ This shows that the system is not much concerned about personnel" [51]^ (p1161−1162)^ . The limited information nurses received from their organisation about the disease and how this coupled with their fear and anxiety was also revealed [54].

Category two of this meta-synthesis identified various challenging environmental factors within the organisation that affected nurses' psychological well-being. For example, experiencing psychological pain from delivering unpleasant news [54], working in a new context with a new role [53] and behavioural discrimination within the management's practices. As one nurse argues, "Doctors are dominant here. Doctors are given the best gear, but it isn't like that for nurses. A nurse is condemned to work with any equipment they are given" [51] ^(p1164)^.

Findings in category three describe how the experience of social support and social pressure were varied amongst nurses. For instance, nurses face stigmatisation from society who consider them to be more vulnerable to contracting an infection [59]. Besides, nurses also have to confront escalating public fear caused by spreading misinformation about the disease. As highlighted in the statement, "Fear was evident on the faces of the patients and their families. There were rumours that any patient who was hospitalised would definitely die" [52]^ (p 575)^. Conversely, participants revealed that these negative experiences also include meaningful interactions such as increased social solidarity and altruism, recognition and gratitude from family, colleagues and patients [53, 62].

Category four sheds light on the lack of PPE as a consistent challenge for all nurses. A fact which negatively impacted their safety [59], as one participant reported, "They don't easily provide the [protective] gear for us" [51] ^(p1162)^ . Another aspect to consider is the physical exhaustion and the difficulties they encountered with regard to PPE during providing care [57].  Besides these barriers, extended usage of PPE while caring for Covid-19 patients had resulted in significant issues, such as spots and skin damage as illustrated by one nurse, "We are truly tired. In this ward, all female nurses are covered in spots because of stress, and some have hormonal disorders. Our skin is badly damaged under the mask and medical caps"  [51] ^ (p1164)^.

### Meta synthesis three: additional aspects compounding negatively on nurses’ well-being

Three categories were revealed in this meta-synthesis: [1] the fear of contracting an infection affected nurses' psychological well-being and their life outside of their clinical role, [2] the psychological needs and concerns of nurses were not addressed, and [3] professional turnover intention.

Findings in category one indicates that the interactions of nurses with the Covid-19 patients greatly influenced their social lives outside the hospital. Some nurses expressed their anxiety about the possibility of carrying the disease at home, which drove them to consider self-isolation at home [59, 51, 54]. While another nurse highlights the impact of Covid-19 on their social life and how it affected them emotionally as cited in the statement, "My social relationships have decreased a lot, I cannot see my friends, my best friend was supposed come visit me after a month, but those in the unit, where my friend worked, said that he/ she could not visit Nurse 9. My friend came to me really demoralized. He/she did not tell anyone about his/her visit. When he/she returned, he/she acted as if he/she had not visited me. This situation wears me down emotionally" [58]^ (p163)^ . Furthermore, the loss of a loved one to the disease had a profound effect on their emotional well-being [62].

The findings in category two substantially influenced their well-being, with authority support, respect and value being urgent needs. Participants felt they were not treated as they deserved, as exhibited in the statement, "I am there for you… Not only the doctor who is there and if you are going to thank, if you really want to thank, do not present it just one person, see me too… We want to be more visible" [61]^ (p1369)^.

Further, the need for psychosocial support as well as addressing their concerns was also emphasised by nurses as an urgent need [58].

On the other hand, findings in category three found that nurses became alienated from the profession as a reflection of the above-mentioned obstacles [51]. This was illustrated by nurses when they shared, "I became very alienated from the nursing profession. I mean, I am discouraged by how the profession is regarded… I’ve decided to quit the profession" [61]^ (p137)^.

### Meta-synthesis four: nurses show maturity during Covid-19

This meta-synthesis included three categories: [1] different psychosocial and behavioural resilience approaches were adopted by nurses, [2] commitment to the nursing profession and [3] advancing in nursing practice.

In contrast to the challenges mentioned above, findings in category one describes the psychological adjustment and resilience adopted by nurses to alleviate the negative experience, as commented in the statement, "I was trying to comfort myself by saying over and over again that 1 day all this will end, and we will return to normal life, even in my hardest times" [62] ^(p 9)^ . Whereas in other studies, nurses avoided concentrating on their negative experiences and avoided watching any news to maintain their mental well-being  [58] interestingly, some nurses have modified their diets to boost their immune systems against the virus [53].

The findings in category two identifies different positive aspects that nurses viewed as adding value to their professions. Many felt obligated to deliver care as they viewed it as their responsibility, with one nurse saying, "Let’s say it is work ethics. I know this is the job I have to do. That’s what keeps me going. After all, I have been trained for this. we are on the field in this process who will take care of the patients once we retreat" [58]^(p164)^. Another aspect is that some nurses stated that despite working in threatening situations, they felt like heroes and were satisfied with their vocation; they reported that their sense of dedication was strengthened during this challenging time [62].

In category three, the majority of nurses emphasised an increasing and advance in their roles and responsibilities during the pandemic. They were gaining new knowledge and skills outside of their official roles, as revealed by one nurse, "Before [the pandemic], it was always physicians who greeted the patients first, did triage and gave information about their diseases to patients and their relatives. Now we do these too" [62]^ (p 6)^ . Others highlight that during the pandemic, they realised their inner strength and matured, which they believe will benefit them in their professional nursing roles [61].

### Confidence in review findings

A key aspect of the review process is identifying confidence in the synthesised findings. Accordingly, 'ConQual' has been implemented as the JBI institute proposed implementing it as a method of grading each finding to ensure accuracy and transparency in reporting (see Table 3) [44]. Chapter two provides information on the development of 'ConQual'.

**Table 3:** ConQual summary of finding

**Systematic review title:** Middle Eastern nurses’ views/experiences of work and well-being with the support measures during past disease outbreaks and COVID-19: A qualitative systematic review					
**Population**: Nurses in in-patient settings during Covid-19 and past disease outbreaks such as severe acute respiratory syndrome (SARS), Middle East respiratory syndrome (MERS), and H1N1 influenza					
**Phenomena of interest:** Nurses' perspectives and experiences with support measures during the COVID-19 and past disease outbreaks					
**Context:** Hospitals in Middle East region					
**Synthesised Finding**	**Type of research**	**Dependability**	**Credibility**	**ConQual Score**	**Comments**
1. Nurses experienced challenges during MERS, yet different strategies were implemented by leaders and nurses to manage these challenges	Qualitative—High	Remains unchanged	Remains unchanged	High	**Dependability:** The majority of two primary studies scored 4 or 5 out of 5 for dependability Dependability concerns were that one study had unclear statement locating the researcher culturally/ theoretically **Credibility**: Remain unchanged due to All unequivocal findings U = 17
2. Some well- being support measures were unfulfilled during Covid- 19	Qualitative- High	Remains unchanged	Downgraded one level	Moderate	**Dependability:** The majority of nine primary studies scored 4 or 5 out of 5 for dependability Dependability concerns were that two study the influence of the researcher on the research, and vice- versa, were unclear **Credibility:** Downgraded one level due to a mix of unequivocal and equivocal findings U = 41, C = 7
*3.* Additional aspects compounding negatively on nurses’ well- being	Qualitative—High	Remains unchanged	Downgraded one level	Moderate	**Dependability:** The majority of nine primary studies scored 4 or 5 out of 5 for dependability Dependability concerns were that two study the influence of the researcher on the research, and vice- versa, were unclear **Credibility:** Downgraded one level due to a mix of unequivocal and equivocal findings U = 18, C = 1
4. Nurses show maturity during Covid- 19	Qualitative—High	Remains unchanged	Downgraded one level	Moderate	**Dependability:** The majority of seven primary studies scored 4 or 5 out of 5 for dependability Dependability concerns were that two study the influence of the researcher on the research, and vice- versa, were unclear **Credibility:** Downgraded one level due to a mix of unequivocal and equivocal findings. U = 17, C = 5

## Discussion

In this review, the synthesised findings are discussed in depth to obtain greater insight into the identified issues, to identify implications and to make recommendations that can help nursing practices elevate the nursing workforce and improve nurse well-being during future pandemics.

### Synthesis one: nurses experienced challenges during MERS, yet different strategies were implemented by leaders and nurses to manage these challenges

This review highlighted that the lack of support measures (e.g. organisational, social and psychological support) during the MERS pandemic did not hinder leaders’ and nurses’ efforts to implement additional support measures during MERS pandemic to alleviate strain and sustain their well-being. Contrary to expectations, the findings of this review revealed that nurses who experienced a lack of adequate support measures during MERS also experienced a similar lack of support measured during COVID-19.

A recent systematic review also found a remarkable similarity between the past pandemics and COVID-19, although the settings and phenomena of interest differed [63]. However, contrary to what was reported in a systematic review by Billings [63] the present review identified a few crucial differences between these pandemics in terms of their utilisation of additional support measures. Therefore, even if the obstacles introduced by these pandemics were roughly comparable, each global epidemic had its own influence on well-being support measures.

**Fig. 3:**
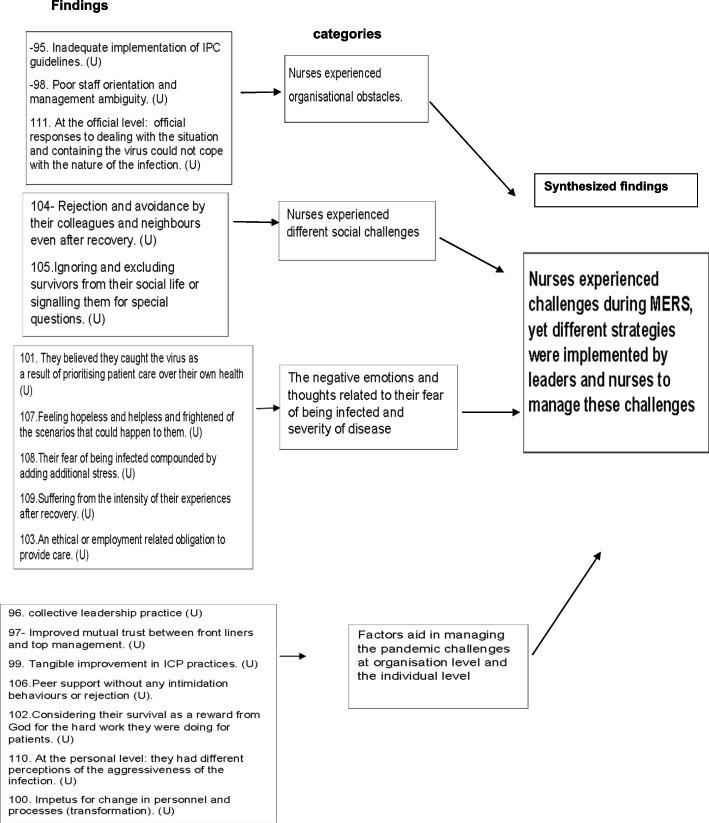
Summary of findings related to meta-synthesis one

The impacted support measures were aggravated by many difficulties nurses experienced, such as social stigmatisation and rejection, the psychological burden associated with disease and the fact that the organisational responses were not commensurate with the intensity of the pandemic (Fig. 3). In the present findings, poor organisational support led nurses to demand clear communication and consistency of information; these desired support measures were also cited in another study [64].

Contrary to the findings of the present review concerning the paucity of support measures during MERS, other research in a similar region in the Middle East argued that a well-established management plan was utilised to assist nurses and facilitate the management of the MERS outbreak [65]. However, the occurrence of perceived support and the inadequacy of support measures vary according to the preparedness of each organisation. This could explain the discrepancy between the findings of Al-Dorzi [65] and the findings of this review.

**Fig. 4:**
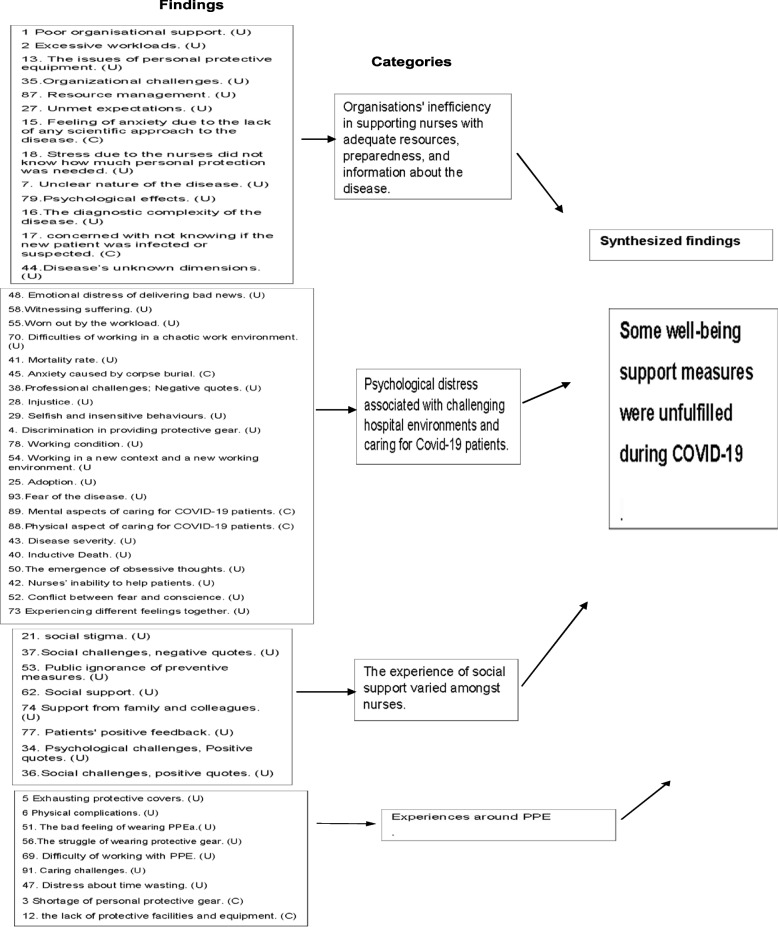
Summary of findings related to meta-synthesis two

On the flipside, leaders’ acknowledgement of their nurses’ well-being was essential to an effective response to the MERS outbreak (Fig. 3). This was not the case in the Covid-19 pandemic (Fig. 4). This is what this review revealed as the most remarkable difference between how support measures were implemented in the two pandemics. Furthermore, the review findings shed light on other support measures that have been adopted by leaders and nurses to alleviate the above-mentioned challenges, such as building trust and promoting teamwork, effective leadership styles, resilience behaviours and peer support. Some of these factors are in line with what has been proposed in the literature [24, 66]. This synthesis seems to provide new insight into how nurses’ well-being can be acknowledged in future pandemics, as these support measures contribute favourably to nurses’ well-being (Fig. 3).

### Synthesis two: some well-being support measures were unfulfilled during COVID-19

This meta-synthesis revealed that support measures during Covid-19 were either unfilled or not applied sufficiently. This may be due to the unprecedented scope of the COVID-19 pandemic, or it may be that the rapidly evolving situation impeded efforts to focus on well-being support measures. This view is also supported by Young and Fick [67]  who reported that when the Covid-19 crisis occurred, the predominant focus was on expanding the capacity of healthcare services to accommodate and treat Covid-19 patients.

Of the unfulfilled support measures, nurses reported that the lack of organisational support in various areas and the insufficiency in personal protective equipment (PPE) had the greatest impact on their well-being. In contrast, although social support varied among nurses, it positively impacted their psychological well-being. It is likely that some of the challenges that created stress and obstacles in prior pandemics occurred again during the Covid-19 pandemic but, as explored in the review findings (Fig. 4), with more significant obstacles related to the work environment. Such as, delivering bad news to the families and behavioural discrimination within the management's practices. Some of these obstacles were also detected in another study during Covid-19 [68] but were not detected in previous pandemics [66, 69].

PPE shortages were reported not only in the Middle East but worldwide [70]. However, the present findings differ from other studies globally and reveal some crucial insights into nurses’ use of PPE in the Middle East, highlighting distinct obstacles to their practice that are rarely discussed in the literature.

Nurses and doctors in the Middle East experience a notable disparity in their social standing; the distribution of authority within the healthcare system tends to favour physicians, whereas nurses are viewed as assistants instead of professional practitioners [71]. This inequality in authority and treatment can explain the discriminatory behaviour regarding nurses’ PPE that has been highlighted in this review. However, this finding cannot be generalised to the entire Middle East, since only two studies in this review addressed this discrimination.

Nevertheless, nurses’ dissatisfaction with being categorised as inferior to doctors and their efforts to ensure their concerns are known are not new, and the findings of this review further illuminate this issue. These finding are in opposition to Billings [63] systematic review, which reported that various healthcare professionals’ present experiences do not differ. However, the findings of this study make it clear that the difficulties faced by nurses are indeed different from those of other healthcare professionals.

The constant inadequacy of support measures identified in this review and in another study from a wider geographical area [72], clarifies that the lack of well-being support measures is not due to religious or cultural influences. However, the studies included in this review tended not to consider how religion or culture affected the adaptability of these measures in crises.

A possible explanation for these results may be that these measures may not have responded to implementation barriers that associated with each pandemic challenges, as this review explores the recurrent and continued psychological distress associated with lack of support measures. This interpretation is also consistent with explanation from broader literature [23].

Moreover, it seems possible that the Covid-19 pandemic exposed the vulnerability of medical and public health institutions in sustaining nurses’ well-being to respond to global pandemic. Therefore, in light of the nurses’ experiences covered in this review, learning from this synthesis finding seems essential for healthcare organisations to empower the well-being for effective response to crisis.

### Synthesis three: additional aspects negatively impacting nurses’ well-being

**Fig. 5:**
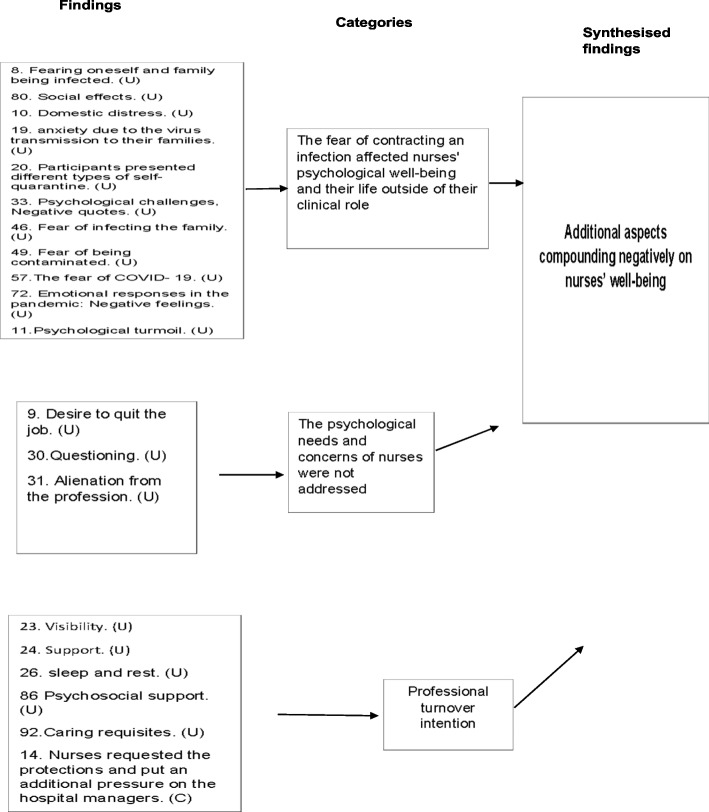
Summary of findings related to meta-synthesis three

To gain a better understanding about nurses' well-being during Covid-19 and determine whether they have faced other psychological burdens or obstacles during the Covid-19 pandemic, this review also consider further exploration. In this study, nurses identified additional aspects on their workplace and personal lives that contributed to unsustainable well-being or compounded their negative experiences (Fig. 5).

Additional challenges for nurses included coping with the uncertainty and stress induced by the unknown nature of the virus and the potential to infect others, which forced some nurses to self-quarantine at home. In particular, they distanced themselves from their family and friends. This ultimately had a considerable influence on their social lives outside their clinical roles, causing domestic distress and resulting in loneliness, which aggravated the other barriers the nurses had to cope with. This conclusion is supported by similar findings in the wider literature [73].

Furthermore, it was revealed in this review that nurses initially felt overwhelming fear and anxiety, but feelings of frustration followed shortly after. Nurses perceived that despite the threats they faced, the obligations they met and the effort they put into their work, they were not being valued and respected by their organisation when disclosing their primary needs and concerns. This finding echoes the findings of another study conducted in Wuhan [74] and is aligned with the broader literature.

This provides evidence for the findings of this review by highlighting the fact that nurses’ psychological needs and concerns are sometimes misunderstood or inadequately addressed.

However, the findings of the current review do not support Billings [63] finding that nurses felt valued because their organisations recognised their safety and supported them. These differences can be explained by the fact that nurses’ perceptions of value, support and their needs may differ. For example, in a wider geographical area, nurses reported that their needs included adequate information as well as interpersonal and family needs [75]; others reported needing adequate training with psychological preparation [76]. In the present review, the need for nurses to be visible, recognised and appreciated and the demand for psychosocial support and resource management were the most prominent needs (Fig. 5).

This review revealed that some nurses intend to leave their jobs, which may be due to the aforementioned unfulfilled support measures and needs. However, the nurses in some of the studies included in this review were interviewed at the end of their shifts, which may have exacerbated their desire to quit their jobs due to their feelings of fatigue. Yet, the alienation from nursing profession has been reported widely [77]. A better understanding of these needs will allow nurses to be offered support that is in line with their reported preferences and viewpoints.

### Synthesis four: nurses showed maturity during Covid-19

Resilience is defined as an individual’s capacity to recover from challenging situations in their lives and to effectively overcome obstacles [78]. Despite the challenges noted above, this review suggests that psychological adjustment and resilience were significant in helping nurses overcome unfulfilled support measures and alleviating the burden of Covid-19. Furthermore, this finding illuminates the strength with which nurses tackled the crisis and the process by which they matured in their professions.

**Fig. 6:**
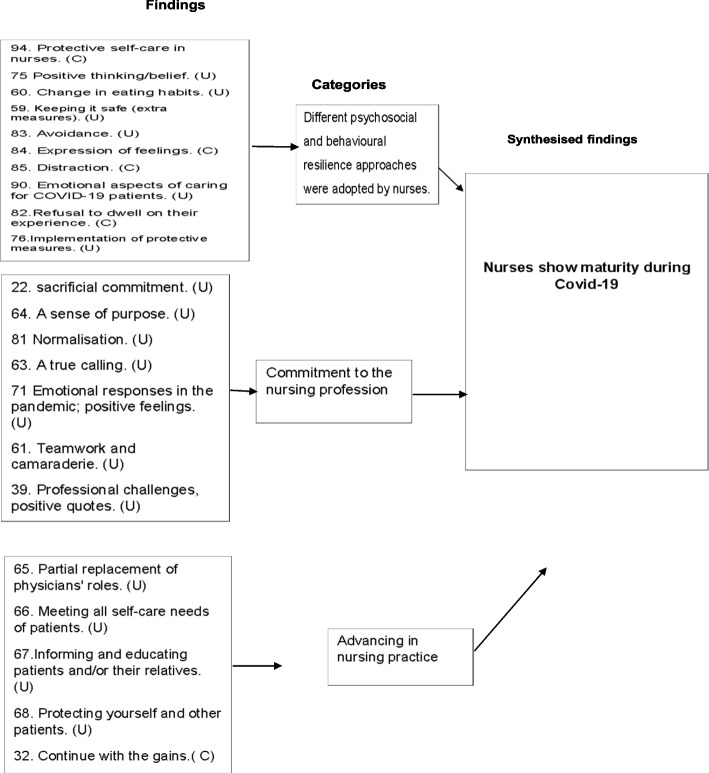
Summary of findings related to meta-synthesis four

Therefore, while this review has revealed that some nurses were traumatised by pandemic consequences (Fig. 4 and Fig. 5), for others, it was an opportunity to increase self-awareness regarding their health, overcome their fear and advance in their nursing practice (Fig. 6). This finding emphasises the significance of resilience in preserving nurses’ mental well-being and fostering personal development.

In line with the review findings, nurses also relied on coping mechanisms to ease the strain they encountered in their work environments during earlier pandemics. For example, during the SARS pandemic, nurses shifted their mindsets and strived to be optimistic rather than frightened [79]. During the MERS pandemic, having a sense of humour, sharing jokes with co-workers and engaging in relaxing activities helped alleviate nurses’ tension [36]. Interestingly, in this review, fear of Covid-19 encouraged nurses to develop defensive behaviours, such as avoiding following the news about Covid-19 and modifying their eating habits to boost their immune systems.

In summary, nurses’ preferred coping strategies may vary based on their unique perceptions of risk or the availability of support sources. What is not yet clear in this review is the impact of religious or spiritual beliefs on providing comfort or stress relief. The included studies tended not to address this issue, which indicates the need for further research on this topic.

What distinguishes the current findings from those of other studies is that some nurses showed a sense of professional commitment despite the threats involved in nursing practice during the Covid-19 pandemic. In contrast, during the SARS outbreak in Japan, nurses were found to be unwilling or hesitant to care for infected patients [80].

Likewise, during the MERS outbreak in South Korea, nurses felt that they were under pressure related to their commitment to care for infected patients [81]. This review, which focused on Middle Eastern nurses suggests that it is possible that nurses’ intentions to care for infected patients are associated with their religious or spiritual beliefs. This view was echoed by another study in Saudi Arabia [82].

This review also demonstrates that advancement within the nursing profession was possible during Covid-19 pandemic, as some nurses reported experiencing learning and professional development (Fig. 6). This finding suggests that resilience not only mitigates the impact of a pandemic on nurses’ mental health but may also serve as a mediator in fostering clinical performance. Wider literature supports this interpretation [83].

### Implications of the review findings and recommendations

The following recommendations for practice are graded based on the JBI Grades of Recommendation [84]. Recommendations are graded as either Grade A (strong recommendation) or Grade B (conditional recommendation) see Appendix 9 in Additional file 1. The recommendations derived from this meta-synthesis aim to extend the understanding of nurses’ experiences with support measures during the Covid-19 pandemic. Furthermore, they could serve as a reference for nursing managers, policymakers or hospitals that wish to make improvements or modifications to better protect nurses' well-being in future pandemics and to ensure high quality of care.

### Recommendations for practice

1. This review conditionally recommends that, during a pandemic, healthcare systems must establish action plans based on nurses’ valuable experiences. Furthermore, healthcare systems need to be improved by (1) establishing an emergency plan and preparations that aid nurses in responding to future pandemics in a coordinated and effective manner and (2) involving nurses at all stages of guideline development. (Grade B)
2. It is recommended that nurse managers ensure that adequate and equal distribution of safety equipment is treated as a priority. This is necessary to ensure that nurses can practice safe and effective work, to reduce the adverse mental health effects associated with their fear of being infected and to mitigate discriminatory behaviour. (Grade A)
3. This review suggests that nursing staff should be encouraged to feel that they are valued in their institutions. Nurse managers should consider nurses’ needs and concerns, such as their desire to feel visible and recognised and to receive psychosocial support to protect and enhance their well-being. (Grade A)
4. It is conditionally recommended that managers pay close attention to demonstrating their understanding of the burdens nurses face and the value of nurses’ efforts. Managers must also show commitment to creating and maintaining a positive work environment. This could help alleviate the mental distress, pressure and trauma that nurses experience in their work environments. (Grade B)

### Recommendations for research

1. Most studies in the Middle East that have explored nurses’ responses to the pandemic have been cross-sectional studies, and qualitative research has been limited. Moreover, qualitative studies on HCWs during the Covid-19 pandemic have not explored nurses’ experiences as a discrete group. The current literature requires further qualitative research to fill this gap.
2. The generalisability of the findings would be enhanced by a similar review conducted in languages other than English.
3. The guidelines of well-being and additional studies on this issue should extend beyond an emphasis on clinical understandings of well-being to examine employees’ needs and the contextual variables that could impede the implementation of recommendations or hinder support measures.
4. Attention is currently being paid to the impact of the Covid-19 pandemic on frontline nurses’ mental well-being. However, there is still a lack of research into psychosocial interventions focusing on what works for whom and when and how religious or cultural aspects may influence nurses’ resilience or willingness to care. Therefore, there is a need for future studies to explore these topics.
5. The Covid-19 pandemic might have a positive outcome in terms of illuminating nurses’ psychological and behavioural resilience during a crisis; however, it is unclear how nurses' resilience may also serve as a mediator in improving clinical performance. Hence, further studies might be useful to explore this.

### Strengths and limitations of the review

#### Strengths

To the author knowledge, no qualitative systematic review has investigated nurses' perspectives on well-being support measures during prior pandemics and Covid-19 in the Middle East. Therefore, this review findings expand the understanding of support policies considering the current experience, as well as offer novel insights into the study area.

The review synthesis compiled findings from studies on the challenges nurses confront during disease outbreaks; therefore, the results were based on actual experiences of professionals and not on a hypothetical modelled scenario. As a result, the present investigation can be used as a reference for nursing practice, policy development, and future study.

The meta-synthesis also highlighted the scarcity of qualitative studies in Middle Eastern countries. Additional studies from multiple countries in the Middle East are warranted to explore all perspectives on the matter. To develop a comprehensive view about well-being support strategies already in place and whether they need to be reformed in the face of a rapidly evolving crisis.

The review’s protocol was pre-registered on PROSPERO, for publication as agreed between the authors.

The review abstract has been accepted for oral presentation at the 50th global congress on nursing care and research conference in London.

### Limitations

Some of the included studies may have contributed to bias within the review because of the translation of data on nursing personal experiences from local languages to English. However, the involvement of native English speakers in translating the included studies might ease the bias.

Due to the time allocated for the review being limited, the inclusion criteria were circumscribed to studies published in English. Therefore, this review may be subject to publication bias, restricting the ability to generalise the findings to the experiences of all nurses in the Middle East, as most of the data were collected from four countries.

## Conclusions

This systematic review evaluated and aggregated qualitative studies to investigate the previously defined question; “Have the well-being support measures that were implemented in previous pandemics influenced nurses’ well-being during the Covid-19 pandemic in the Middle East”.

Eleven studies were considered, which provided 111 findings, incorporated into 14 categories, and summarised into four meta-syntheses. This meta- synthesis was performed with the aid of the JBI approach to obtain a comprehensive understanding of the phenomenon of interest and generate further recommendations. The ConQual Summary of Findings implies that the level of confidence of the 11 included studies and relevant findings yielded high or moderate results.

The review aimed to investigate four objectives, which included: [1] exploring nurses' perspectives and experiences with support measures during previous pandemics and Covid-19; [2] examining which of the support measures have been altered during Covid-19; [3] understanding if nurses faced unique challenges during Covid-19 to maintain their well- being; [4] surveying what might be different during Covid-19 regarding their well-being and whether their cultural and religious beliefs had any influence on it. Although the review addressed the first three research objectives, it did not delve into the influence of cultural and religious practices on well-being in the geographical context of the Middle East, limitation that needs to be tackled further in future research.

The broader literature research conducted referring to prior pandemics advocated nurses’ leaders and policymakers to develop several support measures in case of future pandemics, to preserve nurses' well-being and quality of care for patients.

This review found that organisation and care facilities in the Middle East followed the same practices; nevertheless, nurses experience burnout and psychological distress through Covid-19 despite these support measures. Contrary to expectations, the syntheses revealed that nurses went through somewhat similar experiences during past pandemics and Covid-19.

A significant difference highlighted in this review between MERS outbreaks and Covid-19 is that an effort was made during MERS outbreak to acknowledge nurses' well-being, whereas in Covid-19 pandemic the officials failed to fulfil staff needs and sufficiently apply some support measures, which led to professional burnout. This could have resulted from the previously recommended support measurements might not considering the barriers of implementation, as Covid-19's unparalleled infection rates and the quickly changing situation may have hindered the efforts to enforce well-being support measures.

Undoubtedly, nurses during Covid-19 face countless psychological difficulties because of different complications, including working in a different context with a new role, inadequate supplies of PPE, psychological stress from delivering unpleasant news, witnessing patients suffering, the fear of contracting the infection and the effects of Covid-19 on their private life outside the clinical role.

Therefore, nurses resort to various coping strategies, safeguarding themselves and their loved ones, while also seeking support from authorities to meet their unfulfilled needs. Notwithstanding, the pandemic has also had uplifting positive outcomes on nurses' professional and personal lives. For example, nurses demonstrated maturity in their perception of the novel threat and care for the infected patient, improving self-awareness of their health and professional commitment with the increased responsibility in their roles.

Although the review aimed to investigate well-being support measures and their influence on nurses during different disease outbreaks, the interview questions in the included qualitative studies were only limited to nurses’ experiences with pandemics. On this account, further studies are needed to include more evidence on which measures were most effective for each group and to understand the contextual variables that impede implementation of recommendations or hinder support measures.

Nurse managers, employers and policymakers in the Middle East would benefit from this review’s findings as a reference and implementing the suggestions brought forward in this meta-synthesis. Providing nurses with sufficient resources, a positive workplace environment, emotional backing, and support networks can assist them in thriving and adapting to stressful contexts.

## Supplementary Information

**Additional file 1.**

## Data Availability

All data generated or analysed during this study are included in this published article [and in the Additional file 1].

## Abbreviations

Covid-19: Coronavirus disease
MERS: Middle East respiratory syndrome
SARS: Severe Acute Respiratory Syndrome
H1N 1: Influenza A Virus Subtype H1N1
ConQual: Confidence In Quality
CINAHL: Cumulative Index to Nursing and Allied Health Literature
PROSPERO: International prospective register of systematic reviews
MEDLINE: Medical Literature Analysis and Retrieval System Online
HCWs: Healthcare workers
JBI: Joanna Briggs Institute
EBP: Evidence-based practice
PPE: Personal Protective Equipment
PICo: Population, Phenomenon of interest and Context
PRISMA: Preferred Reporting Items for Systematic Reviews and Meta-Analyses
JBI-QARI: Qualitative Assessment and Review Instrument
PICo: Population, Phenomenon of interest and Context
WHO: World Health Organization
