# Correction: Middle Eastern nurses’ views/experiences of work and well-being with the support measures during past disease outbreaks and COVID-19: a qualitative systematic review

**DOI:** 10.1186/s12912-024-02429-3

**Published:** 2024-10-10

**Authors:** Sara Ahmed Marair, Nigel Slater

**Affiliations:** 1 King Saud Medical City, Riyadh, Saudi Arabia; 2 University of Nottingham, Nottingham, NG1 5NT UK

**BMC Nursing (2023) 22:230**

10.1186/s12912-023-01343-4

It was brought to the authors attention that some sections in this paper may be perceived to overlap with another dissertation by one of University of Nottingham’s MSc students. The previous dissertation was submitted prior to the authors’ work and publication of this paper. After careful consideration and liaison with the Student and Head of Research Integrity, the authors agreed that the student should not be deprived the advantage of their work which is unpublished at the time of publication of this Correction article; therefore, the authors have decided to revise the focus of this paper by making the following amendments:

1) The title should instead read as follows: “Results of Qualitative Systematic review on the implementation of well-being support measures that were recommended during previous pandemics (H1N1, MERS, and SARS) and comparing it to the COVID-19 pandemic in the Middle East.”

2) The Objectives sub-section of the Abstract should instead read as follows: “To investigate the implementation of well-being support measures during previous pandemics and comparing it with COVID-19 pandemic in the Middle East).”

3) The authors state that the focus of the manuscript is instead to be considered about addressing and discussing the synthesised findings of this systematic review, ensuring that it does not overlap with the aforementioned dissertation, and ensuring no disadvantage to or undermining of the student’s and our work.

