# Supplementary material for: Middle Eastern nurses’ views/experiences of work and well-being with the support measures during past disease outbreaks and COVID-19: a qualitative systematic review
Source: BMC Nurs. 2023 Jul 3;22:230. doi: 10.1186/s12912-023-01343-4 (PMC10316637; doi:10.1186/s12912-023-01343-4)
Supplement: Supplementary file 1 — Additional file 1. [file 12912_2023_1343_MOESM1_ESM.docx]

**[Table of Contents](#_bookmark84)**

[[Appendix 1: Search strategy and the search keywords 1](#_bookmark84)](#_Toc119845335)

[[Appendix 2: JBI tool to perform critical appraisal for qualitative study 6](#_bookmark84)](#_Toc119845336)

[[Appendix 3: JBI-QARI data extraction tool for qualitative research](#_bookmark84)^[1](#_bookmark84)^ [17](#_bookmark84)](#_Toc119845337)

[[Appendix 4: The JBI Approach to qualitative synthesis (Meta-aggregation)](#_bookmark84) ^[2](#_bookmark84)^ [19](#_bookmark84)](#_Toc119845338)

[[Appendix 5: ConQual summary](#_bookmark84)^[1](#_bookmark84)^ [20](#_bookmark84)](#_Toc119845339)

[[Appendix 6: Excluded studies after reading full text 21](#_bookmark84)](#_Toc119845340)

[[Appendix 7: Characteristics of included studies 24](#_bookmark84)](#_Toc119845341)

[[Appendix 8: The findings extracted from the included studies 29](#_bookmark84)](#_Toc119845342)

[[Appendix 9: JBI Grades of Recommendation](#_bookmark84) ^[28](#_bookmark84)^ [68](#_bookmark84)](#_Toc119845343)

# Appendix 1: Search strategy and the search keywords

**Search conducted in CINAHL (EBSCO) on 30 November 2021**

| **Search number** | **Search term** | **Records retrieved** |
| --- | --- | --- |
| **#1** | nurs* OR “nurs* staff “OR "nurs* hospital" OR “staff nurs*”  OR MH "Registered Nurses" | (1,979,708) |
| **#2** | MH wellbeing OR well-being OR well being OR MH “quality of life” OR MH “mental health”  OR resilienc* | (1,346,291) |
| **#3** | “Protective factor” OR “psychological intervention” OR  “protective measures” | (26,962) |
| **#4** | experiences OR perceptions OR attitudes OR views OR  feelings | (3,268,886) |
| **#5** | burnout OR burn-out OR burn out OR stress OR MH “occupational stress” OR MH “compassionate fatigue” OR MH “mental distress” OR MH “psychological distress” OR MH  “emotional distress” | (1,371,743) |
| **#6** | MH H1N1 OR MH “swin flu”  OR MH “swine influenza” OR “influenza” | (47,589) |
| **#7** | mers OR MH “middle east  respiratory syndrome” | 21,804 |
| **#8** | MH "SARS Virus" OR MH  "Severe Acute Respiratory Syndrome" | (10,504 |

| **#9** | MH covid-19 or MH coronavirus or MH 2019-ncov or MH sars-cov-2 or “corona  virus disease” | (353,226) |
| --- | --- | --- |
| **#10** | MH “middle eastern” OR “middle east” OR israel or iraq OR iran OR saudi arabia OR Lebanon OR turkey OR iran  OR united arab emirates | (745,762) |
| **#11** | S1 AND S2 AND S3 AND S4  AND S5 AND S6 AND S7 AND S8 AND S9 | (103,633) |
| **#12** | #11 AND #10 – English and  from 2003 till 2021- Middle east | (10) |

**Search conducted in MEDLINE (Ovid) on 30 November 2021**

| **Search number** | **Search term** | **Records retrieved** |
| --- | --- | --- |
| **#1** | Influenza A Virus, H3N2 Subtype/ or Influenza A Virus, H1N1 Subtype/ or Influenza A virus/ or  h1n1.mp. | 307184 |
| **#2** | MERS.mp. or Middle East Respiratory Syndrome  Coronavirus/ | 7864 |
| **#3** | Severe Acute Respiratory  Syndrome/ OR SARS Virus/ | 5696 |
| **#4** | covid 19 OR coronavirus/ OR  SARS-COV2/ | 388212 |

| **#5** | (past OR previous) pandemic OR  epidemic OR "disease outbreak"/ OR infectious disease/ | 2742351 |
| --- | --- | --- |
| **#6** | nurses/ OR nurse specialists/ OR  Nursing Staff, Hospital/ | 2261053 |
| **#7** | Mental Health/ OR well-being.mp. OR "Quality of Life"/ OR Adaptation, Psychological/ or  Resilience/ | 6697808 |
| **#8** | stress, psychological/ OR burnout, psychological/ OR burnout, professional/ OR emotions/ OR psychological  distress/ | 2913374 |
| **#9** | middel east/ OR Saudi Arabia OR Egypt OR Iraq OR Yemen or Lebanon OR Oman OR Kuwait or Qatar OR Bahrain OR Syria OR  Jordan OR United Arab Emirates | 280361 |
| **#10** | #1 AND #2 AND #3 AND #5 AND #6 AND #7 AND #8 AND #9.  limited 2003-2021, English | 608 |
| **#11** | #4 AND #6 AND #7 AND #8 AND  #9 - English and from 2003 till 2021 | 272 |

**Search conducted in NUsearch on 30 November 2021**

| **Search number** | **Search term** | **Records retrieved** |
| --- | --- | --- |
| **#1** | ( H1N1 OR influenza) AND Covid-19 AND (mers OR “middle east respiratory syndrome”) AND "Severe Acute Respiratory Syndrome"  AND “Qualitative study” AND (nurse* OR "staff nurs*”) AND (experiences OR perceptions OR experienced OR Views) AND (burnout OR “psychological distress” OR “mental distress” AND (“mental health” OR “quality of life” OR well*being OR resilienc* OR “protective factor” ) AND  “middle east” | 48 |
| **#2** | #1 limited 2003 till 2021-  English | 48 |

**Search conducted in Google scholar on 30 November 2021**

| **Search number** | **Search term** | **Records retrieved** |
| --- | --- | --- |
| **#1** | "Qualitative study" AND Eastern Mediterranean region AND experience AND health care workers AND (past OR previous) AND pandemic. From 2003 till 2021- peer  reviewed | **725** |

**The search keywords with the different databases**

| **Key concept** | **Synonyms/ related terms/alternative forms for key words** |
| --- | --- |
| **Nurse** | “nurs*” OR "clinic*nurse" OR "hospital nurs*" OR "Front line  nurs*” OR "staff nurse*" OR nursing care OR "Nursing health professional" |
| **COVID-19** | "covid 19" OR coronavirus OR SARA virus OR COVID-19 OR  2019-ncov OR sars-cov-2 OR cov-19 |
| **Previous pandemic** | “past” OR "pandemic*" OR "epidemic*" OR "disease outbreak" OR "infectious disease" OR "H1N1" OR "influenza type A" OR "influenza virus" OR "influenza A virus" OR "SARS epidemic" OR "H1N1 pandemic" OR "MERS outbreak" OR h1n1 OR "swine flu" OR "swine influenza" OR "sars 2003" OR mers OR "middle east respiratory  syndrome" |
| **Burnout** | burnout or "professional burnout" or stress or "psychological distress" or "mental distress" or turnover OR “mental distress” OR burn-out OR burn out OR “occupational stress”  OR “compassionate fatigue” OR “emotional distress” |
| **Wellbeing** | "mental health" or "quality of life" or "wellbeing" or resilience OR “well*being” OR resiliency OR resilient OR “protective  factor of wellbeing” OR “psychological intervention” |
| **Middle east** | "Middle east region" OR "Middle east" or Saudi Arabia or Egypt or Iraq or Yemen or Lebanon or Oman or Kuwait or Qatar or Bahrain or Syria or Jordan or United Arab Emirates  OR "middle eastern studies" OR israel OR iran |

# Appendix 2: JBI tool to perform critical appraisal for qualitative study

**JBI Critical Appraisal Checklist for Qualitative Research**

| Reviewer |  |  |  |  | Date |
| --- | --- | --- | --- | --- | --- |
| Author: | Moradi et al. | Year: | 2021 | Record Number: | 1 |

|  | Yes | No | Unclear | Not  applicable |
| --- | --- | --- | --- | --- |
| 1. Is there congruity between the stated  philosophical perspective and the research methodology? | yes |  |  |  |
| 2. Is there congruity between the research  methodology and the research question or objectives? | yes |  |  |  |
| 3. Is there congruity between the research  methodology and the methods used to collect data? | yes |  |  |  |
| 4. Is there congruity between the research methodology and the representation  and analysis of data? | yes |  |  |  |
| 5. Is there congruity between the research  methodology and the interpretation of results? | yes |  |  |  |
| 6. Is there a statement locating the  researcher culturally or theoretically? | yes |  |  |  |
| 7. Is the influence of the researcher on the  research, and vice- versa, addressed? | yes |  |  |  |
| 8. Are participants, and their voices,  adequately represented? | yes |  |  |  |
| 9. Is the research ethical according to current criteria or, for recent studies, and is there evidence of ethical  approval by an appropriate body? | yes |  |  |  |
| 10. Do the conclusions drawn in the research report flow from the analysis,  or interpretation, of the data? | yes |  |  |  |

Overall appraisal: 10/10 Include yes □Exclude □Seek further info □Comments (Including reason for exclusion): It is funded but it is relevant to my inclusion criteria

**JBI Critical Appraisal Checklist for Qualitative Research**

Reviewer Date

Author: Sadati et al. Year: 2021 Record Number: 2

- 1. Is there congruity between the stated philosophical perspective and the research methodology?
  2. Is there congruity between the research methodology and the research question or objectives?
  3. Is there congruity between the research methodology and the methods used to collect data?
  4. Is there congruity between the research methodology and the representation and analysis of data?
  5. Is there congruity between the research methodology and the interpretation of results?
  6. Is there a statement locating the researcher culturally or theoretically?
  7. Is the influence of the researcher on the research, and vice- versa, addressed?
  8. Are participants, and their voices, adequately represented?
  9. Is the research ethical according to current criteria or, for recent studies, and is there evidence of ethical approval by an appropriate body?
  10. Do the conclusions drawn in the research report flow from the analysis, or interpretation, of the data?

Yes No Unclear Not applicable

Yes

Yes

Yes

Yes

Yes Yes Yes Yes

Unclear

Unclear

Overall appraisal: 9/10 Include yes □ Exclude □ Seek further info □ Comments (Including reason for exclusion):

**JBI Critical Appraisal Checklist for Qualitative Research**

Reviewer Date

Author: Yıldırım, Aydoğan and Bulut Year: 2021 Record Number: 3

|  | Yes | No | Unclear | Not  applicable |
| --- | --- | --- | --- | --- |
| 1. Is there congruity between the stated philosophical perspective and the  research methodology? | yes |  |  |  |
| 2. Is there congruity between the research methodology and the research question  or objectives? | yes |  |  |  |
| 3. Is there congruity between the research methodology and the methods used to  collect data? | yes |  |  |  |
| 4. Is there congruity between the research methodology and the representation  and analysis of data? | yes |  |  |  |
| 5. Is there congruity between the research  methodology and the interpretation of results? | yes |  |  |  |
| 6. Is there a statement locating the  researcher culturally or theoretically? | yes |  |  |  |
| 7. Is the influence of the researcher on the  research, and vice- versa, addressed? | yes |  |  |  |
| 8. Are participants, and their voices,  adequately represented? | yes |  |  |  |
| 9. Is the research ethical according to current criteria or, for recent studies, and is there evidence of ethical approval  by an appropriate body? | yes |  |  |  |
| 10. Do the conclusions drawn in the  research report flow from the analysis, or interpretation, of the data? | yes |  |  |  |

Overall appraisal: 10/10 Include: yes □ Exclude □ Seek further info □

Comments (Including reason for exclusion)

**JBI Critical Appraisal Checklist for Qualitative Research**

Reviewer Date

Author: Chegini et al. Year: 2021 Record Number: 4

|  | Yes | No | Unclear | Not  applicable |
| --- | --- | --- | --- | --- |
| 1. Is there congruity between the stated philosophical perspective and the  research methodology? | yes |  |  |  |
| 2. Is there congruity between the research methodology and the  research question or objectives? | yes |  |  |  |
| 3. Is there congruity between the research methodology and the  methods used to collect data? | yes |  |  |  |
| 4. Is there congruity between the research methodology and the  representation and analysis of data? | yes |  |  |  |
| 5. Is there congruity between the  research methodology and the interpretation of results? | yes |  |  |  |
| 6. Is there a statement locating the  researcher culturally or theoretically? | yes |  |  |  |
| 7. Is the influence of the researcher on  the research, and vice- versa, addressed? | yes |  |  |  |
| 8. Are participants, and their voices,  adequately represented? | yes |  |  |  |
| 9. Is the research ethical according to current criteria or, for recent studies, and is there evidence of ethical  approval by an appropriate body? | yes |  |  |  |
| 10. Do the conclusions drawn in the research report flow from the analysis,  or interpretation, of the data? | yes |  |  |  |

Overall appraisal: 10/10 Includeyes □ Exclude □ Seek further info □

Comments (Including reason for exclusion): It is funded but it is relevant to my inclusion criteria

**JBI Critical Appraisal Checklist for Qualitative Research**

Reviewer Date

Author: Galehdar et al. Year: 2020 Record Number 5

|  | Yes | No | Unclear | Not  applicable |
| --- | --- | --- | --- | --- |
| 1. Is there congruity between the stated philosophical perspective and the  research methodology? | yes |  |  |  |
| 2. Is there congruity between the research methodology and the  research question or objectives? | yes |  |  |  |
| 3. Is there congruity between the research methodology and the  methods used to collect data? | yes |  |  |  |
| 4. Is there congruity between the research methodology and the  representation and analysis of data? | yes |  |  |  |
| 5. Is there congruity between the  research methodology and the interpretation of results? | yes |  |  |  |
| 6. Is there a statement locating the  researcher culturally or theoretically? | yes |  |  |  |
| 7. Is the influence of the researcher on  the research, and vice- versa, addressed? | yes |  |  |  |
| 8. Are participants, and their voices,  adequately represented? | yes |  |  |  |
| 9. Is the research ethical according to current criteria or, for recent studies, and is there evidence of ethical  approval by an appropriate body? | yes |  |  |  |
| 10. Do the conclusions drawn in the research report flow from the analysis,  or interpretation, of the data? | yes |  |  |  |

Overall appraisal: 10/ 10 Includeyes □ Exclude □ Seek further info □

Comments (Including reason for exclusion): It is funded but it is relevant to my inclusion criteria

**JBI Critical Appraisal Checklist for Qualitative Research**

Reviewer Date

Author: Villar et al. Year: 2021 Record Number: 6

|  | Yes | No | Unclear | Not  applicable |
| --- | --- | --- | --- | --- |
| 1. Is there congruity between the stated philosophical perspective and the  research methodology? | yes |  |  |  |
| 2. Is there congruity between the research methodology and the  research question or objectives? | yes |  |  |  |
| 3. Is there congruity between the research methodology and the  methods used to collect data? | yes |  |  |  |
| 4. Is there congruity between the research methodology and the  representation and analysis of data? | yes |  |  |  |
| 5. Is there congruity between the  research methodology and the interpretation of results? | yes |  |  |  |
| 6. Is there a statement locating the  researcher culturally or theoretically? | yes |  |  |  |
| 7. Is the influence of the researcher on  the research, and vice- versa, addressed? | yes |  |  |  |
| 8. Are participants, and their voices,  adequately represented? | yes |  |  |  |
| 9. Is the research ethical according to current criteria or, for recent studies, and is there evidence of ethical  approval by an appropriate body? | yes |  |  |  |
| 10. Do the conclusions drawn in the research report flow from the analysis, or interpretation, of the  data? | yes |  |  |  |

Overall appraisal: 10/10 Include yes □ Exclude □ Seek further info □

Comments (Including reason for exclusion): It is funded but it is relevant to my inclusion criteria

**JBI Critical Appraisal Checklist for Qualitative Research**

Reviewer Date

Author: Kandemir, Yılmaz and Sönmez Year: 2021 Record Number 7

|  | Yes | No | Unclear | Not applicable |
| --- | --- | --- | --- | --- |
| 1. Is there congruity between the stated philosophical perspective and the research methodology? | yes |  |  |  |
| 2. Is there congruity between the research methodology and the research question or objectives? | yes |  |  |  |
| 3. Is there congruity between the research methodology and the methods used to collect data? | yes |  |  |  |
| 4. Is there congruity between the research methodology and the representation and analysis of data? | yes |  |  |  |
| 5. Is there congruity between the research methodology and the interpretation of results? | yes |  |  |  |
| 6. Is there a statement locating the researcher culturally or theoretically? | yes |  |  |  |
| 7. Is the influence of the researcher on the research, and vice- versa, addressed? | yes |  |  |  |
| 8. Are participants, and their voices, adequately represented? | yes |  |  |  |
| 9. Is the research ethical according to current criteria or, for recent studies, and is there evidence of ethical approval by an appropriate body? | yes |  |  |  |
| 10. Do the conclusions drawn in the research report flow from the analysis,  or interpretation, of the data? | yes |  |  |  |

Overall appraisal: 10/10 Include yes □ Exclude □Seek further info □

**JBI Critical Appraisal Checklist for Qualitative Research**

Reviewer Date

Author: Kackin et al. Year: 2021 Record Number 8

|  | Yes | No | Unclear | Not  applicable |
| --- | --- | --- | --- | --- |
| 1. Is there congruity between the stated philosophical perspective and  the research methodology? | yes |  |  |  |
| 2. Is there congruity between the research methodology and the  research question or objectives? | yes |  |  |  |
| 3. Is there congruity between the research methodology and the  methods used to collect data? | yes |  |  |  |
| 4. Is there congruity between the research methodology and the  representation and analysis of data? | yes |  |  |  |
| 5. Is there congruity between the  research methodology and the interpretation of results? | yes |  |  |  |
| 6. Is there a statement locating the researcher culturally or  theoretically? | yes |  |  |  |
| 7. Is the influence of the researcher on the research, and vice- versa,  addressed? | Unclear |  |  |  |
| 8. Are participants, and their voices,  adequately represented? | yes |  |  |  |
| 9. Is the research ethical according to current criteria or, for recent studies, and is there evidence of ethical  approval by an appropriate body? | yes |  |  |  |
| 10. Do the conclusions drawn in the research report flow from the analysis, or interpretation, of the  data? | yes |  |  |  |

Overall appraisal: 9.5/10 Include: yes □ Exclude□ Seek further info

Comments (Including reason for exclusion): The researchers were acquainted with six of the participants**,** no methods were provided to address interviewer bias.

**JBI Critical Appraisal Checklist for Qualitative Research**

Reviewer Date

Author: Almutairi et al. Year: 2018 Record Number: 9

|  | Yes | No | Unclear | Not  applicable |
| --- | --- | --- | --- | --- |
| 1. Is there congruity between the stated philosophical perspective and the  research methodology? | yes |  |  |  |
| 2. Is there congruity between the research methodology and the  research question or objectives? | yes |  |  |  |
| 3. Is there congruity between the research methodology and the  methods used to collect data? | yes |  |  |  |
| 4. Is there congruity between the research methodology and the  representation and analysis of data? | yes |  |  |  |
| 5. Is there congruity between the  research methodology and the interpretation of results? | yes |  |  |  |
| 6. Is there a statement locating the  researcher culturally or theoretically? |  |  | unclear |  |
| 7. Is the influence of the researcher on  the research, and vice- versa, addressed? | yes |  |  |  |
| 8. Are participants, and their voices,  adequately represented? | yes |  |  |  |
| 9. Is the research ethical according to current criteria or, for recent studies, and is there evidence of ethical  approval by an appropriate body? | yes |  |  |  |
| 10. Do the conclusions drawn in the research report flow from the analysis,  or interpretation, of the data? | yes |  |  |  |

Overall appraisal: 9.5/ 10 Include: yes □ Exclude □ Seek further info

Comments (Including reason for exclusion): Firstly, the statement for the author participant on the research process was only stated clearly on the data analysis process. In addition, the study was funded. But it is relevant to my inclusion criteria.

**JBI Critical Appraisal Checklist for Qualitative Research**

Reviewer Date

Author: Al Knawy et al. Year: 2019 Record Number: 10

|  | Yes | No | Unclear | Not  applicable |
| --- | --- | --- | --- | --- |
| 1. Is there congruity between the stated philosophical perspective and  the research methodology? | yes |  |  |  |
| 2. Is there congruity between the research methodology and the  research question or objectives? | yes |  |  |  |
| 3. Is there congruity between the  research methodology and the methods used to collect data? | yes |  |  |  |
| 4. Is there congruity between the  research methodology and the representation and analysis of data? | yes |  |  |  |
| 5. Is there congruity between the research methodology and the  interpretation of results? | yes |  |  |  |
| 6. Is there a statement locating the  researcher culturally or theoretically? | yes |  |  |  |
| 7. Is the influence of the researcher on  the research, and vice- versa, addressed? | yes |  |  |  |
| 8. Are participants, and their voices, adequately represented? | yes |  |  |  |
| 9. Is the research ethical according to current criteria or, for recent studies, and is there evidence of ethical approval by an appropriate body? | yes |  |  |  |
| 10. Do the conclusions drawn in the research report flow from the analysis, or interpretation, of the  data? | yes |  |  |  |

Overall appraisal: 10/ 10 Include: yes □ Exclude □Seek further info Comments (Including reason for exclusion): Although the participant addressed adequately, however, the quotes does not state is it for the doctors or nurses.

**JBI Critical Appraisal Checklist for Qualitative Research**

Reviewer Date

Author: Kheirandish et al. Year: 2020 Record Number: 11

|  | Yes | No | Unclear | Not  applicable |
| --- | --- | --- | --- | --- |
| 1. Is there congruity between the stated philosophical perspective  and the research methodology? | yes |  |  |  |
| 2. Is there congruity between the research methodology and the  research question or objectives? | yes |  |  |  |
| 3. Is there congruity between the research methodology and the  methods used to collect data? | yes |  |  |  |
| 4. Is there congruity between the research methodology and the representation and analysis of  data? | yes |  |  |  |
| 5. Is there congruity between the research methodology and the  interpretation of results? | yes |  |  |  |
| 6. Is there a statement locating the  researcher culturally or theoretically? | yes |  |  |  |
| 7. Is the influence of the researcher on the research, and vice- versa,  addressed? |  |  | Unclear |  |
| 8. Are participants, and their voices,  adequately represented? | yes |  |  |  |
| 9. Is the research ethical according to current criteria or, for recent studies, and is there evidence of ethical approval by an appropriate  body? | yes |  |  |  |
| 10. Do the conclusions drawn in the research report flow from the analysis, or interpretation, of the  data? | yes |  |  |  |

Overall appraisal: 9.5/10 Include yes □ Exclude □Seek further info □

# Appendix 3: JBI-QARI data extraction tool for qualitative research^1^


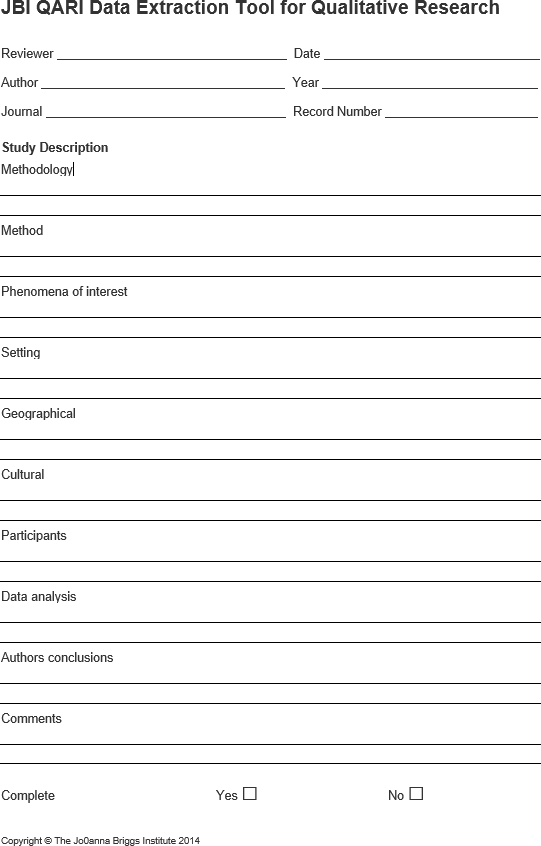


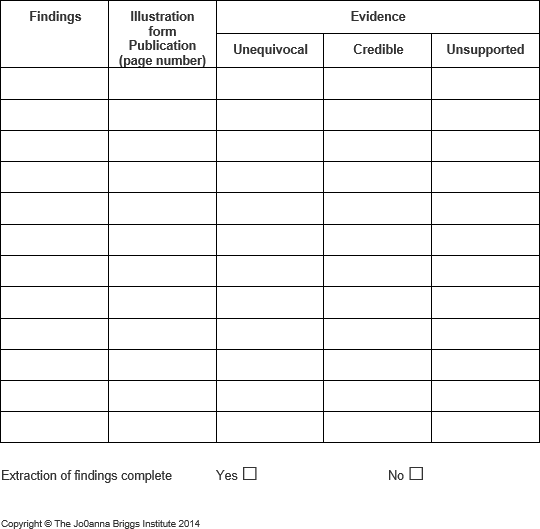


# Appendix 4: The JBI Approach to qualitative synthesis (Meta-aggregation) ^2^


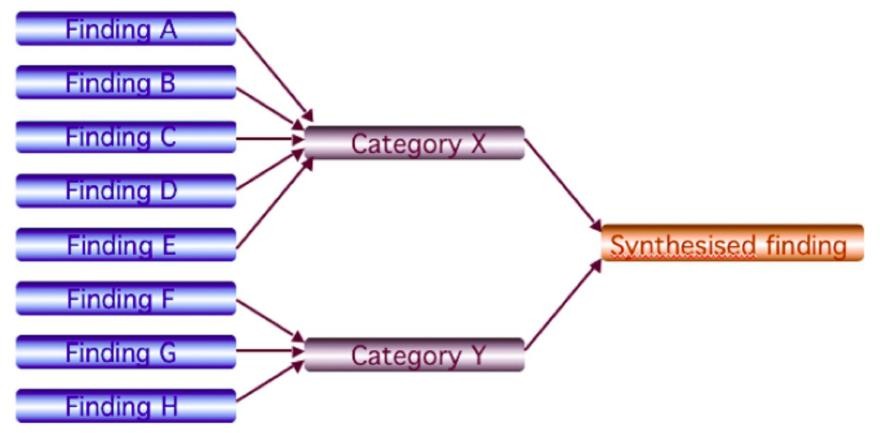


# Appendix 5: ConQual summary^1^


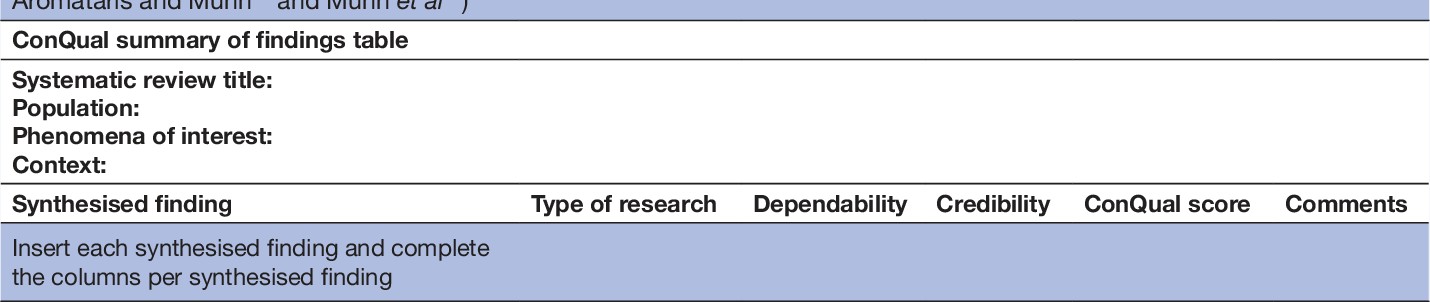


# Appendix 6: Excluded studies after reading full text

| **Studies** | **Reason for**  **exclusion** |
| --- | --- |
| Sarikaya O, Erbaydar T. Avian influenza outbreak in Turkey through health personnel's views: a qualitative study. BMC Public Health. 2007 Dec;7(1):1. | Only one  nurse included.^3^ |
| Alnazly E, Khraisat OM, Al-Bashaireh AM, Bryant CL. Anxiety, depression, stress, fear and social support during COVID-19 pandemic among Jordanian healthcare workers. PloS one. 2021;16(3):0247679. | This study adopted a cross- sectional.^4^ |
| Kim SC, Sloan C, Chechel L, Redila M, Ferguson J. Severe Burnout and Poor Mental Health Among Healthcare Workers 6 Months After COVID-19 Pandemic Declaration: What Can We Learn for Future Emergencies?. J Nurs Adm. 2021;51(11):554–60. https://nusearch.nottingham.ac.uk/permalink/f/t1e4u0/TN_cdi_proquest_miscellaneous_2585912053. Accessed 10 July 2022. | A cross- sectional survey.^5^ |
| Pourvakhshoori N, Norouzi K, Ahmadi F, Hosseini M, Khankeh H. Nurse in limbo: A qualitative study of nursing in disasters in Iranian context. PloS one. 2017;12(7):0181314. | It is explored nurses who had experience at the time of natural  disasters.^6^ |
| El‐Monshed AH, Amr M, Ali AS, Elmasry YM, Zoromba M. Nurses' knowledge, concerns, perceived impact and preparedness toward COVID‐19 pandemic: A cross‐sectional survey. Int J Nurs Pract. 2021;27(6):13017. | This study is cross- sectional study.^7^ |

| Danielis M, Peressoni L, Piani T, Colaetta T, Mesaglio M, Mattiussi E, et al. Nurses’ experiences of being recruited and transferred to a new sub‐intensive care unit devoted to COVID‐19 patients. J Nurs Manag. 2021;29(5):1149–58. | This study describes the experiences of Italian  Nurses.^8^ |
| --- | --- |
| Kang HS, Son YD, Chae S, Corte C. Working experiences of nurses during the Middle East respiratory syndrome outbreak. Int J Nurs Pract. 2018;24(5):12664. | This study was in South Korea.^9^ |
| Khalid I, Khalid TJ, Qabajah MR, Barnard AG, Qushmaq IA. Healthcare workers emotions, perceived stressors and coping strategies during a MERS-CoV outbreak. Clin Med Res. 2016;14(1):7–14. | Cross- sectional descriptive survey  design.^10^ |
| Baker OG, Alamri AA, Aboshaiqah AE. A descriptive study to analyse the disaster preparedness among Saudi nurses through self‐regulation survey. J Nurs Manag. 2019;27(7):1479–84. | It employed quantitative descriptive design.^11^ |
| Bahrami M, Aliakbari F, Aein F. Iranian nurses' perception of essential competences in disaster response: A qualitative study. J Educ Health Promot. 2014; 3:81. https://nusearch.nottingham.ac.uk/permalink/f/t1e4u0/TN_cdi_pubmedcentral_primary_oai_pubmedcentral_nih_gov_4165091. Accessed 10 July 2022. | This study about natural disasters.^12^ |
| Alsubaie S, Hani Temsah M, Al-Eyadhy AA, Gossady I, Hasan GM, Al-Rabiaah A, et al. Middle East Respiratory Syndrome Coronavirus epidemic impact on healthcare workers' risk perceptions, work and personal lives. J Infect Dev Ctries. 2019;13(10):920–6. | This was a cross- sectional survey.^13^ |

| Bukhari EE, Temsah MH, Aleyadhy AA, Alrabiaa AA, Alhboob AA, Jamal AA, et al. Middle east respiratory syndrome coronavirus (MERS-CoV) outbreak perceptions of risk and stress evaluation in nurses. J Infect Dev Ctries. 2016;10(8):845–50. | Questionnair e survey using statistical analysis.^14^ |
| --- | --- |
| Karabulak H, Kaya F. The Relationship Between Psychological Resilience and Stress Perception in Nurses in Turkey During the COVID-19 Pandemic. Nurs Res.2021;29(6):175. | Cross- sectional design.^15^ |
| Shahrour G, Dardas LA. Acute stress disorder, coping self‐efficacy and subsequent psychological distress among nurses amid COVID‐19. J Nurs Manag. 2020;28(7):1686–95. | Quantitative, cross- sectional.^16^ |

# Appendix 7: Characteristics of included studies

| **Study** | **Country and Pandemic** | **Methodology and methods** | **Phenomena of interest and settings** | **Description of main results** |
| --- | --- | --- | --- | --- |
| (Al Knawy et al., 2019).^17^ | Saudi Arabia  MERS | Qualitative methods of process evaluation  Semi- structured individual interviews and focus groups. | Decision-makers and clinical staff working in tertiary healthcare centre.  Included 10 nurses who were in direct patient care contact with MERS-CoV patients during  the outbreak. | Lack of organisational preparedness to apply infection control measures, low employee morale, and high anxiety hampered management progress. However, the development of trust and teamwork and the utilisation of collaborative leadership all contributed to effective management of pandemic challenges. |
| (Almutairi et al., 2018).^18^ | Saudi Arabia  MERS | Qualitative research approach.  Face-to-face semi- structured interview. | Frontline healthcare providers among them 4 nurses who survived from the disease infection and have experienced with MERS patient. | The experiences of the participants were broken down into four Them:  caring for others in the defining moments, perceived prejudice behaviours and stigmatization, lived moments of traumatic fear and despair, and denial and underestimation of the seriousness of the disease at the individual and organiational levels. |
| (Chegini et al., 2021).^19^ | Iran Covid-19 | A qualitative phenomenolo gical design.  Semi‐ structured interviews were carried out either via face‐to‐face or tele- phone. | 15 nurses who provided care for patients infected by COVID‐19 in Iran's public hospitals. | Caregiving for patients infected with COVID19 presented nurses with several difficulties, which were broken down into four Them: psychological (eight subthemes), organisational (six), social (six), and professional (five subthemes). According to the current classification, psychological, social, and professional obstacles have positive and negative consequences, but organisational challenges have  exclusively negative effects. |

| **Study** | **Country and Pandemic** | **Methodology and methods** | **Phenomena of interest and settings** | **Description of main results** |
| --- | --- | --- | --- | --- |
| (Galehdar et al., 2020).^20^ | Iran Covid-19 | Qualitative study.  Semi- structured in- depth telephone interviews. | 20 nurses caring for patients with COVID-19. | This study revealed that nurses who cared for COVID-19 patients experienced a significant amount of psychological burden. The causes of such distress were the mortality of patients, the unknown dimensions of the disease, the workplace environment, professional obligations, and individual  characteristics. |
| (Kackin et al., 2021).^21^ | Turkey Covid-19 | Descriptive phenomenolo gical approach.  Interviews were face-to- face via the internet. | 10 nurses who cared for patients diagnosed with COVID-19. | Three main themes emerged from the experiences and psychosocial challenges faced by nurses caring for patients with COVID-19. Which are ‘effects of the outbreak’, ‘short- term coping strategies’ and ‘needs’. |
| (Kalateh- Sadati et al., 2021).^22^ | Iran Covid-19 | Qualitative study.  Semi structured interviews. | 24 nurses who worked at hospitals specified for COVID-19. | It was identified that all the participants had encountered challenges and negative experiences. No one had sufficient knowledge of the new virus or how to resist it. In this instance, the most significant experiences were associated with inadequate preparedness, the highest perceived risk, family protection,  stigmatisation, and professional commitment. |

| **Study** | **Country and Pandemic** | **Methodology and methods** | **Phenomena of interest and settings** | **Description of main results** |
| --- | --- | --- | --- | --- |
| (Kandemir, Yılmaz and Sönmez, 2021).^23^ | Turkey Covid-19 | Qualitative, using a phenomenolo gical approach.  Semi- structured interview was conducted only once via WhatsApp video call. | 12 emergency nurses were working in the emergency department. | This study reported that emergency nurses during the pandemic encountered an increase in their roles in a more chaotic setting.The analysis of the data revealed four themes: “Increasing roles and responsibilities as an emergency nurse”, “Difficulties of working in pandemic conditions”, “Emotional responses in the pandemic”  and “Strategies for coping with the effects of the pandemic” |
| (Kheirandish et al., 2020).^24^ | Iran Covid-19 | Qualitative conventional content analysis  In-depth  semi-structure face-to-face interviews. | 10 nurses participated working in the COVID-19 ward. | Providing care for covid-19 patients has presented several issues for nurses. As a result, four themes were identified: COVID-19 nurses’ experiences of caring, multiple aspects of caring for COVID-19 patients, the challenges and requisites of COVID-19 care, the fear of being infected by the virus and the nurses’ protective self-care |
| (Moradi et al., 2021).^25^ | Iran Covid-19 | Qualitative descriptive approach.  Semi- structured face- to- face interviews. | 17 nurses working in intensive care units. | Nurses caring for COVID- 19 patients reported facing four difficulties: "organisation's inadequacy in assisting nurses," "physical fatigue," "living with unknown," and "psychological strain due to the disease". |

| **Study** | **Country and Pandemic** | **Methodology and methods** | **Phenomena of interest and settings** | **Description of main results** |
| --- | --- | --- | --- | --- |
| (Villar et al., 2021).^26^ | Qatar Covid-19 | Qualitative, phenomenolo gical design.  A semi- structured interview done face to face. | 30 registered nurses who took care of patients with COVID- 19. | Frontline nurses experienced numerous problems that had a negative impact on their physical, emotional, and mental health. The study yielded three main themes:  Challenges of working in a COVID- 19 facility, surviving COVID- 19 and Resilience of  Nurses. |
| (Yıldırım, Aydoğan and Bulut, 2021).^27^ | Turkey Covid-19 | Descriptive phenomenolo gical approach.  Semi- structured interviews were carried out individually via Skype. | 17 front- line nurses caring for coronavirus- infected patients. | Four major themes have originated: "needs", "angry”, “questioning" and "decision". Among these requirements are the need to be seen and supported, to adapt, and to have enough sleep and rest.  The nurses were frustrated because their needs were not satisfied, they felt they were mistreated, and they were subjected to acts of selfishness and insensitivity. They began to doubt their chosen career and had to decide whether to  withdraw from it. |

# Appendix 8: The findings extracted from the included studies

Moradi, Y., Baghaei, R., Hosseingholipour, K., and Mollazadeh, F. (2021) Challenges experienced by ICU nurses throughout the provision of care for COVID‐19 patients: A qualitative study. *Journal of Nursing Management, 29*(5), 1159-1168.

| **Finding** | **Illustration from publication**  **(page number)** | **Evidence** | | |
| --- | --- | --- | --- | --- |
| **Theme 1: Organisation's inefficiency in supporting nurses**  1 Poor organisational support | "We expect officials to come and visit us, motivate us, and boost our morale. Since the outbreak of Coronavirus, no university deputies or hospital managers have come to ask “What are you doing here? What kinds of problems are you facing?” This shows that the system is not much concerned about personnel".  (P16, male nurse) pp.1161-1162 | Unequivocal (U)  **U** | Credible (C) | Not Supported (NS) |
| 2 Excessive workload | "The shifts we work are really killing, not tiresome, but killing". "We have not been given leave, and we  They have been told that we must be in the hospital during this crisis. They don't give us proper off time, either".  (P14, female nurse) p.1162. | **U** |  |  |
| 3 Shortage of personal protective gear | "They don't easily provide the  [protective] gear for us". (P13, female nurse) p.1162. |  | **C** |  |

| 4 Discrimination in providing protective gear | "Doctors are dominant here. Doctors are given the best gear, but it isn't like that for nurses. A nurse is condemned to work with any equipment they are given".  (P17, female nurse) p.1164. | **U** |  |  |
| --- | --- | --- | --- | --- |
| **Themes 2:**  **Physical exhaustion**  5 Exhausting protective covers | "The clothes we wear make us very tired during the shift.  Besides, with these on, we cannot eat or use the bathroom, especially during night shifts.  Pardon me, many women have UTI, and some suffer from constipation because they have sluggish bowel". (P12, female nurse) p.1164.  "The heaviness of protective covers and gears reduces our abilities. It is very hard to focus on work with these clothes"  (P4, male nurse) p.1164. | **U** |  |  |
| 6 Physical complications | "We are truly tired. In this ward, all female nurses are covered in spots because of stress, and some have hormonal disorders. Our skin is badly damaged under the mask and medical caps" (P5,  female nurse) p.1164. | **U** |  |  |

| **Them 3:**  **Living with uncertainty Psychological**  7. Unclear nature of the disease | "The biggest concern is the lack of knowledge about this disease, since there is no treatment for it and you don't know the prognosis. You know what is going to happen with many diseases. For instance, with the flu, you get well in 10 days, but you don't really know what happens with this disease. You don't know its clinical picture either. Are fever, cough, and shortness of breath the actual signs or not? You don't really know. We have had many of such patients with none of these signs. One patient said that he only had diarrhea. It's been four days that I've had diarrhea myself" (P11,  male nurse) p.1164. | **U** |  |  |
| --- | --- | --- | --- | --- |
| 8. Fearing oneself and family being infected | "We are worried about our loved ones, and fear that we might be accidentally infected and carry this unknown disease home and pass on the disease to the spouse, children, father, and  mother".(P1, male nurse) p.1165. | **U** |  |  |
| 9. Desire to quit the job | "It is not clear how long this situation could last and what is going to happen. We were all unhappy about being a nurse, and wish we had another job that  would take us away from this | **U** |  |  |

|  | setting"(P8, female nurse)  p.1165. |  |  |  |
| --- | --- | --- | --- | --- |
| **Them4: Psychological burden of the disease**  10. Domestic distress | "Our lives have gone off- track, and we have no peace. Before the current pandemic, when we got home after the shift, we could at least cuddle our children. My wife and I would at least talk together, but not now! We cannot cuddle our children. Contacts are limited. We suspect and doubt anything and every- thing in our own home, which is the safest place in terms of Corona"  (P16, male nurse) p.1165. | **U** |  |  |
| 11. Psychological turmoil | "The stress caused by this disease has made me a little more aggressive, as I sometimes even become hostile toward my family, especially my brother "  (P15, female nurse) p.1165. | **U** |  |  |

Kalateh Sadati, A., Zarei, L., Shahabi, S., Heydari, S., Taheri, V., Jiriaei, R., and Lankarani, K. (2021) Nursing experiences of COVID‐19 outbreak in Iran: A qualitative study. *Nursing Open, 8*(1), 72-79.

| **Finding** | **Illustration from publication**  **(page number)** | **Evidence** | | |
| --- | --- | --- | --- | --- |
| **Theme 1: defected preparedness**  12. the lack of  protective facilities and equipment. | "We have to be so safe. It's unclear if the patient is coming is infected or not, can we respond to him/her just based on a simple temperature?" (Sahar, Qazvin)  p .75. | Unequivocal (U) | Credible (C)  C | Not Supported (NS) |
| 13. The issues of personal protective equipment | "I always thought that the equipment would be perfect in the face of such a crisis. However, under the limita- tion of required prerequisites, you have to work in this environment with the least  precision"(Marzie, Qazvin) p.75. | U |  |  |
| 14. Nurses requested the protections and put an additional pressure on the hospital managers | "I believed that N95 mask is not needed for everyone. I tell my staffs there is no need to even mask for an or- dinary patient, but they do not agree. In this situation, no one trusts anything"  (a head nurse, Shiraz) p. 75. |  | C |  |
| **Them2:**  **The worst perceived risk**  15. Feeling of anxiety due to the lack of any scientific approach to  the disease | "When we were told that our hospital was going to have a corona ward in Arak, I didn't understand the crisis and couldn't figure out what was going to hap- pen to us."  (Zahra, Arak) p.75. |  | **C** |  |

| 16.The diagnostic complexity of the disease | "Now, it seems that you are going through a dark room, you don't know anything and it's stressful. You don't know anything until the light comes on. This is what we feel in the face of Coronavirus "  (Mohammed, Qazvin) p. 75 | U |  |  |
| --- | --- | --- | --- | --- |
| 17. concerned with not knowing if the new patient was infected or suspected | "Dr. Alavi was admitted for respiratory distress in our ward for a week. He used to talk to us using a cell phone. There was a close connection between the staff and him. Although he often wore a mask, it was later revealed that he  was infected"(Sara, Arak) p. 75 |  | **C** |  |
| 18. Stress due to the nurses did not know how  much personal  protection was needed | "I was anxious due to the ambiguity of personal pro- tection. A doctor comes with masks and gloves and another one puts on no  mask or a glove" (Ali Qazvin) p.75 | U |  |  |
| **Them3:**  **family protection**  19. anxiety due to the virus transmission to their families | "The more concern I had was for my family. Among my colleges, there were those whose families had a serious illness and were more concerned." (Zahra, Arak) p.  76. | **U** |  |  |
| 20. Participants presented different types of self-quarantine | "At the other side of the yard, we have a warehouse, I told my mom to clean up there for me to quarantine myself"  (Nurse, Qazvin) p. 76. | **U** |  |  |

|  | "In the house, I took all my dishes away from my family and even slept away from my sister"  (Zahra, Qazvin) p. 76 |  |  |  |
| --- | --- | --- | --- | --- |
| **Them4:**  21. social stigma | "Two of our colleague were going to the hospital. One of them got in a taxi and the driver asked him where she was going, when she told him to get her to the hospital, he asked her to get off. The same happened to another colleague when a driver, after knowing her destination –our hospital- did not allow her to get in the taxi"  (a head Nurse, Shiraz) p. 76. | **U** |  |  |
| **Them5:**  22. sacrificial commitment | "Look, if I don't want to come, who will come. Who will care for people? These are our citizens.  We have a professional  commitment and responsibility" (Zahra, Arak) p .76. | **U** |  |  |

Yıldırım, N., Aydoğan, A., and Bulut, M. (2021) A qualitative study on the experiences of the first nurses assigned to COVID‐19 units in Turkey. *Journal of Nursing Management, 29*(6), 1366-1374.

| **Finding** | **Illustration from publication**  **(page number)** | **Evidence** | | |
| --- | --- | --- | --- | --- |
| **Them1: Needs**  23. Visibility | "I am there for you… Not only the doctor who is there and if you are going to thank, if you really want to thank, do not present it just one person, see me too… We want to  be more visible"( P4) p.1369. | Unequivocal (U)  U | Credible (C) | Not Supported (NS) |
| 24. Support | "We needed executives who could understand and manage us, we felt very lonely. Everyone was locked in their room, I didn ' t get much support, frankly, we felt like we were left to our destiny "(P13)  p. 1370. | U |  |  |
| 25. Adoption | "You adapt to one place, then you have to forget about it completely and adapt to the work of the other  place "(P15) p. 1370 | U |  |  |
| 26. sleep and rest | "There has been a tremendous disruption in my sleeping patterns. This is the biggest impact on me  since March"(P5) p. 1370. | U |  |  |
| **Theme 2: Anger**  27. Unmet expectations | "would expect the profession to be paid the importance it should be because we save lives, we make people survive and letting people hold on life"(P3) p .1370. | U |  |  |

|  | "I would expect our managers to spend time with us in the field. Even if they do not help, let them support us, motivate us. But they didn’t"  (P10) p. 1370. |  |  |  |
| --- | --- | --- | --- | --- |
| 28. Injustice | "It was bad because I knew that many of my friends like me were given in clinics in such an unfair way, so it was a bad start "(P4) p.  1370. | U |  |  |
| 29. Selfish and insensitive behaviours | "I had a hard time with these teams, whose members were selfish  and who thought “let someone else take care of the patient rather than  me" (P2) p. 1370. | U |  |  |
| **Theme 3:**  30.Questioning | "I look at it from an economic perspective and then from an emotional perspective, I run into a contradiction. I position myself some- where, but I cannot do the same for this profession, or I position this profession somewhere, but I cannot do the same for myself. Frankly, thoughts began to come to my mind as to whether I would be happy in the future, would this  satisfy me in the future "(P3) p.1371 | U |  |  |
| **Theme 4: Decision**  31. Alienation from the profession | "I became very alienated from the nursing profession. I mean, I am discouraged by how the profession is regarded… I’ve decided to quit  theprofession" (P10) p.1371 | U |  |  |

| 32. Continue with the gains | "Data on the number of patients recovering every day is released, I really thought that it was my contribution to this picture in the number of patients recovered, that this was one part of my jobs, so I felt like a part  of a great success "(P5) p,1371 |  | C |  |
| --- | --- | --- | --- | --- |

Chegini, Z., Arab‐Zozani, M., Rajabi, M., and Kakemam, E. (2021) Experiences of critical care nurses fighting against COVID‐19: A qualitative phenomenological study. *Nursing Forum (Hillsdale), 56*(3), 571-578.

| **Finding** | **Illustration from publication (page**  **number)** | **Evidence** | | |
| --- | --- | --- | --- | --- |
| **Them1:**  33. Psychological challenges, Negative quotes: | "At the time of the outbreak and while caring for our patients, our greatest concern was the stress of getting infected by the virus" (P1) p. 575. | Unequivocal (U)  **U** | Credible (C) | Not Supported (NS) |
| 34. Psychological challenges, Positive quotes: | "My brother posted a photo of me on his Instagram page, and this  sense of pride encouraged me" (P8)  p.575 | **U** |  |  |
| **Theme 2:**  35.Organizational challenges | "The existing protocols were not well observed, and on the other  hand, new protocols were issued every day, which sometimes contradicted the previous ones and  made us doubt "(P10) p. 575 | **U** |  |  |

| **Theme 3:**  36.Social challenges Challenges  Positive quotes: | "People in the community thanked us, and their good prayers  were encouraging and inspiring" (P3)  p. 575. | **U** |  |  |
| --- | --- | --- | --- | --- |
| 37.Social challenges Challenges Negative quotes: | "Fear was evident on the faces of the patients and their families.  There were rumors that any patient  who was hospitalized would definitely die" (P7) p. 575. | **U** |  |  |
| **Theme 4:**  38.Professional challenges Negative quotes: | "The working shift program is not suitable at all… I work for more than 72 hours and cannot sleep for 2 hours yet" (P11) p. 575. | U |  |  |
| 39. Professional challenges  Positive quotes: | "I never intended to leave my job; instead I tried harder" (P11) p. 575. | U |  |  |

Galehdar, N., Kamran, A., Toulabi, T., and Heydari, H. (2020) Exploring nurses' experiences of psychological distress during care of patients with COVID- 19: A qualitative study. *BMC Psychiatry, 20*(1), 489-9.

| **Finding** | **Illustration from publication (page**  **number)** | **Evidence** | | |
| --- | --- | --- | --- | --- |
| **Categorie1:**  Death anxiety  40. Inductive Death | "It is sad that these patients may have been the victims of others’ carelessness " [9] p. 3. | Unequivocal (U)  U | Credible (C) | Not Supported (NS) |
| 41.Mortality rate | "The death of a COVID-19 patient was very stressful for nurses …. especially, the death toll was high at the  beginning …. for example, if we had ten patients, we would have expected 3 death and therefore at least 3 announcement of emergency code in  that shift" [7] p. 3. | U |  |  |
| 42. Nurses’ inability to help patients | "It is agon-izing to see a person deprived of breath, his heart failing, and you can’t do anything about his  suffering it some-times causes me to  feel agitated and distressed and becoming really sad and confused about what I’m going to do?" [10] p .3. | U |  |  |
| **Categorie 2:** Anxiety due to the nature of the disease  43. Disease severity | "Sometimes, a patient  with a brief lung involvement, and who we didn’t expect  at all ..., would die after a while, this was something that  really worried us … we were all  scared"[10] p. 5. | U |  |  |

| 44.Disease’s unknown dimensions | "Not all COVID-19 patients have severe clinical symptoms … there is no correlation between a patient’s death and clinical symptoms … a patient with mild symptoms may die whereas another with severe symptoms  recover … the unknown dimensions of  the disease are numerous " [15] p. 5. | U |  |  |
| --- | --- | --- | --- | --- |
| **Category** 3:  45. Anxiety caused by corpse burial | "It is sad that given the type of their death, families cannot manage to choose the type and place of burial and  even cannot attend the event" [9] p .5. |  | C |  |
| **Category** 4:  46. Fear of infecting the family | "I am more worried about my daughter. She is four years old, … and these children have no understanding of the disease, and you can’t say much to them as it may further cause them a lot of dis-tress, … she is always stuck with me, and I’m so stressed… God  forbid … generally you have a feeling of  guilt that you may infect someone, and that is hard" [11] p. 5. | U |  |  |
| **Categorie** 5:  47. Distress about time wasting | "We even enter the changing room one by one, and that means an hour to just change our clothes at the end of  our shifts... there is a horror … so we couldn’t get to our work on time. For example, while I finished my shift at 1:30 p.m., I would have arrived at home at 2:30 p.m., and it took me until 3 and 4 p.m. to do my work, and I  hadn’t time for the family "[16] p. 6. | U |  |  |

| **Category** 6:  48. Emotional distress of delivering bad news | "We have had mortalities during this period. It was very difficult, especially to inform families ... telling a mother that the condition of her sibling is not good, he is going to die or has died … It’s  really very difficult" [18] p. 6. | U |  |  |
| --- | --- | --- | --- | --- |
| **Category** 7:  49. Fear of being contaminated | "This is a situation that I have to take care of people who may infect me at any moment, and then I may transmit the infection to my family, or the fact that I can even die ...  these thoughts are coming to me … what will happen to my child, to my life  "[10] p. 6. | U |  |  |
| **Category** 8:  50.The emergence of obsessive thoughts | "There has been an obsession with ourselves and our families … there is the same situation at work too, we used to drink tea or water if we had time, but now we can’t "[16] p. 6.  "... I mean, I should wash my hands with every garment, that is, I have to do this constantly for every garment ..."  [15] p. 6. | U |  |  |
| **Category** 9:  51. The bad feeling of wearing PPEa | "These clothes, these excessive precautions that we have to take because of our job, and the nature of the disease we can’t take off or  move our masks, glasses, or shields. It puts pressure on us … when your face itches, and you can’t touch it, this is very annoying these greatly affect  our spirits “ [16] p. 6. | U |  |  |

| **Category** 10:  52. Conflict between fear and conscience | “Along with my fear, I feel pity about why I’m scared and can’t communicate well with patients, it really deters me from communicating with them ....  generally, it’s not a good feeling at all, I feel sad for myself that I have to take care of people who may infect me at any moment, and I may infect my  family, or I may even die" [10] p. 6. | U |  |  |
| --- | --- | --- | --- | --- |
| **Category** 11:  53. Public ignorance of preventive measures | "Why doesn’t the TV show the burial of these patients and the depth of the grave they dig for them, the fact that your family can’t be present at the ceremony, the media should show these to scare people, despite all these rec- ommendations, why don’t people follow and are indiffer- ent to the  guidelines " [10] p. 7. | U |  |  |

Villar, R., Nashwan, A., Mathew, R., Mohamed, A., Munirathinam, S., Abujaber, A., and Shraim, M. (2021) The lived experiences of frontline nurses during the coronavirus disease 2019 (COVID‐19) pandemic in Qatar: A qualitative study. *Nursing Open, 8*(6), 3516-3526.

| **Finding** | **Illustration from publication**  **(page number)** | **Evidence** | | |
| --- | --- | --- | --- | --- |
| **Them1:**  **1-Challenges of working in a COVID- 19 facility**  54. Working in a new context and a new working environment | "The first three weeks I was assigned to test patients for COVID- 19. When the cases increased, I was deployed to the emergency room to care for acute patients. After some time, I was transferred to the inpatient ward. When you are a new staff, you will be trained or at least 3- months. But during the pandemic, I was immediately assigned to a new role. I didn't know a lot about the routine in the In- patient and on my first day I received 5 patients plus admissions and all of them needs critical care. Not to mention I had to activate 3- 4 codes in a shift. It was a struggle"  (N25) pp. 3518- 3520. | Unequivocal (U)  **U** | Credible (C) | Not Supported (NS) |
| 55. Worn out by the workload | "There was an influx of patients. The first two months were difficult. I started having anxiety attacks and sleepless nights. I always thought about  work. I re- called everything | **U** |  |  |

|  | that happened throughout the day, whether I did everything right, gave the right medica- tions, or missed anything"  (N30) p .3520 |  |  |  |
| --- | --- | --- | --- | --- |
| 56. The struggle of wearing protective gear | "Wearing PPE was a struggle, you had to wear and remove it in a certain way. It was hard to breathe, move, and communicate. It was also hot underneath all those layers. It was a hassle to remove the PPEs for a drink. I remembered a time I had to keep my N95 for 3 days. It was for a short while until the supplies were replenished. It was suffocating, but you get  used to it" (N3) p. 3250 | **U** |  |  |
| 57. The fear of COVID- 19 | "I was afraid of contracting the virus. We don't know a lot about it besides the fact that it was a highly contagious virus and people were dying. I have asthma and I know that I will suffer a lot when it gets to me. Worst, I might even be intubated and ad- mitted to intensive care. Both of my parents were in their senior years. I fear for them. I fear that I might bring the virus to  them. Unfortunately, I didn't | **U** |  |  |

|  | have anywhere else to stay so  I had to isolate myself at home"(N15) pp. 3520- 3521 |  |  |  |
| --- | --- | --- | --- | --- |
| 58. Witnessing suffering | "I had three patients who passed away from COVID- 19. We did everything we can. I felt sad for the patients. They don't have any clue what's happening to them because they were sedated. They were powerless. At some point, we tried to wake them up and wean them from the ventilators, but we end up intubating again. I felt sad for their family as well. During the pandemic, visitors were restricted. They were not able to see them or provide them support. There were times that I felt powerless too because I wanted to help the pa- tients,  but I can't"(N7) p. 3521 | **U** |  |  |
| **Them 2:**  **Surviving COVID- 19**  59. Keeping it safe (extra measures) | "Sometimes, I wear double gloves and I place a surgical mask on top of my N95 mask. When I get in the car, I rubbed my hands with alcohol and clean the steering wheel with alcohol. Before entering the house, I take off my shoes and  ask somebody to open the | U |  |  |

|  | door and then I go straight  ahead to the bathroom" (N26) p. 3521 |  |  |  |
| --- | --- | --- | --- | --- |
| 60. Change in eating habits | "I ate a lot during the crisis. I think I gained around 10 kg. I'm exhausted after work; I eat a lot so I can bulk up more energy. I took vitamins more consistently too. I do exercise as well. I limited my alcohol intake during the crisis. I didn't want my immune system to  drop"(N27) p. 3522 | U |  |  |
| 61. Teamwork and camaraderie | "The support was good; no complaints at all. Every now and then, we talked to each other. We were a fam- ily here. You can openly speak or voice out to other charge nurses. I am lucky, I became friends with most of the charge nurses. We were a team. We developed a good relationship. We always looked forward to working together. We developed a camaraderie even though we only met during the COVID- 19 crisis" (N26) p. 3522 | U |  |  |

| 62. Social support | "Talking to someone was the best stress reliever. There was no option to visit your friends or relatives due to this pandemic. Even though it's hard to sit alone in your room after the duty, it was the right thing to do. As much as possible, I will call my wife who's in India to talk about the stress at work. Although sometimes you cannot share everything as it might do more harm than good. So, I call some of my colleagues to whom I am really close to and share my troubles"  (N22) p. 3522 | U |  |  |
| --- | --- | --- | --- | --- |
| **Them 3:**  **Resilience of nurses**  63. A true calling | "I believe as a nurse it is our job to take care of them. I never thought of quitting.  Being a nurse is great be- cause it gives you the capability to help people, re- gardless of who they are and where they come from" (N27)  p. 3522 | U |  |  |
| 64. A sense of purpose | "I never had the idea to resign. It is like giving up on the people who need you and not your career. I feel like I am on a mission. Some of my friends  kept on com- plaining because | U |  |  |

|  | of the restrictions but I go to work to face the patients every single day and I chose to do this" (N7) p. 3522  "On the other hand, there were instances where we felt like heroes when patients survived and improved. I remember one of my patients who went critical. After 2 weeks, he came back to us. He was better. Experiences like this make you feel proud of what  you do"(N20) p. 3522 |  |  |  |
| --- | --- | --- | --- | --- |

Kandemir, D., Yılmaz, A., and Sönmez, B. (2021) Professional and psychological perceptions of emergency nurses during the COVID‐19 pandemic: A qualitative study. *Japan Journal of Nursing Science*, *19*(3), 12470.

| **Finding** | **Illustration from publication**  **(page number)** | **Evidence** | | |
| --- | --- | --- | --- | --- |
| **Theme 1:**  **Increasing roles and responsibilities as an emergency nurse**  65. Partial replacement of physicians' roles | "Before [the pandemic], it was always physi- cians who greeted the patients first, did triage and gave information about their diseases to patients and their relatives. Now we do  these too "(Nurse 10) p. 6. | Unequivocal (U)  U | Credible (C) | Not Supported (NS) |
| 66. Meeting all self-care needs of patients | "Elderly patients who need more care come to the unit, but we cannot take their relatives  with them due to restrictions. | U |  |  |

|  | Patients may wait for a long time for hospitalization or dis- charge in the emergency unit. We are meeting all the self-care needs of these patients "(Nurse  7) p. 6. |  |  |  |
| --- | --- | --- | --- | --- |
| 67.Informing and educating patients and/or their relatives | "In this process, we explained what the patient's condition was, how the process would progress and what kind of a process awaited them and informed the patients and their rela- tives. In this uncertainty, informing patients and their relatives constituted the most impor- tant part of the process "  (Nurse 4) p. 7. | U |  |  |
| 68. Protecting yourself and other patients | "In this period, we also had to protect the other patients against the risk of contamina- tion. Working with protective equipment has made a serious difference in our responsibili-  ties "(Nurse 5) p. 7. | U |  |  |
| **Theme 2:**  **Difficulties of working in pandemic conditions**  69. Difficulty of working with PPE | "My bridge of the nose hurts due to the mask, there is a sensitivity in my nose after the shift, I cannot even touch it.  Double masks or shields cause headaches. Working with protective equipment is really  exhausting.” (Nurse 7) p.7. | U |  |  |

|  | “It makes our job very difficult to intervene with the patient due to the limitation of move- ment in the protective equipment"  (Nurse 1) p. 7. |  |  |  |
| --- | --- | --- | --- | --- |
| 70. Difficulties of working in a chaotic work environment | "The working environment was not physically prepared, there was no distinction between clean and dirty areas, even the dining hall was opposite the COVID-19 polyclinic … With- out any training and psychological support, we - nurses- were directly driven to  the front- line" (Nurse 2) p.7. | U |  |  |
| **Theme 3: Emotional responses in the pandemic**  71 Positive feelings | "I am proud of my profession. I have a very sacred profession as a nurse. It is a pride to be on the frontline in people's most difficult moments, especially in the case of a life-or- death epidemic. Just like in the war… Although I have difficulties from time to time during this period, I'm very glad I'm a nurse …"  (Nurse 7) p. 8. | U |  |  |
| 72 Negative feelings | "I thought that the epidemic could affect my life in some way, but with the fact that I could die, I did not face until our friends in the hos- pital passed away" (Nurse 2) p. 8. | u |  |  |

|  | "Two of my friends passed away in the hospi- tal due to COVID-19, which caused me despair and sadness. At that time, I experi- enced serious fear and anxiety, I never wanted to come to work. I did not have the right to take leave, but I was coming by force …" (Nurse 11)  p. 8. |  |  |  |
| --- | --- | --- | --- | --- |
| 73 Experiencing different feelings together | "In this period, I experience both anxiety, fear, and sometimes happiness… From time to time I can experience all of these mixed feelings  together" (Nurse 6) p. 8. | U |  |  |
| **Theme 4: Strategies for coping with the effects of the pandemic**  74 Support from family and colleagues | "My family supported me the most in this pro- cess. Of course, we tried to support each other with our colleagues" (Nurse 7) p.8  "I think we are a good team, and we have good communication. It is very good to talk to my colleagues during rest hours and to talk about something other than the pandemic. In this process, we work shoulder to shoulder with a full fighting spirit, and we always sup- port each other "  (Nurse 6) p. 9. | U |  |  |

| 75 Positive thinking/belief | "I was trying to comfort myself by saying over and over again that 1 day all this will end, and we will return to normal life, even in my hardest times " (Nurse 10) p. 9.  "I think our profession has a spiritual dimen- sion. During this period, patients need us. For this reason, I start every new day thinking that I have to do my best for them…" (Nurse  8) p .9. | U |  |  |
| --- | --- | --- | --- | --- |
| 76.Implementation of protective measures | "In some periods, I chose to be alone. I was isolating myself from my family, especially after working in the isolation ward. I darkened the bedroom and slept all the time. I was never leaving the room. This was very comforting to me, I believed I could protect my family in this  way …" (Nurse 11) p. 9. | U |  |  |
| 77. Patients' positive feedback | "I know that the patients need us and know that we will not leave them alone in their most difficult times… The patients are at the centre  of our life. They look gratefully,  they pray… This definitely motivates me" (Nurse 3) p. 9. | U |  |  |

Kackin, O., Ciydem, E., Aci, O., and Kutlu, F. (2021) Experiences and psychosocial problems of nurses caring for patients diagnosed with COVID-19 in Turkey: A qualitative study. *International Journal of Social Psychiatry, 67*(2), 158-167.

| **Finding** | **Illustration from publication**  **(page number)** | **Evidence** | | |
| --- | --- | --- | --- | --- |
| **Them1:**  **Effects of the outbreak**  78. Working condition | Nurse 7: "Nurses I have never known or seen. They were assigned to our service unit from another one. I don’t know their reactions . . . we had a dispute the other day with another Nurse . . . It feels as if working in another hospital.  Different patients, a different  order" p. 163 | Unequivocal (U)  **U** | Credible (C) | Not Supported (NS) |
| 79.Psychological effects | Nurse 2: "Uncertainty, . . . really uncertainty about everything . . . what will happen to the hospital, what will happen to us when we go  home" p.163 | U |  |  |
| 80. Social effects | Nurse 9: "My social relationships have decreased a lot, I cannot see my friends, my best friend was supposed come visit me after a month, but those in the unit, where my friend worked, said that he/ she could not visit Nurse  9. My friend came to me really  demoralized . . . He/she did not tell anyone about his/her | U |  |  |

|  | visit . . . When he/she returned, he/she acted as if he/she had not visited me . . . This situation wears me down  emotionally" p.163. |  |  |  |
| --- | --- | --- | --- | --- |
| **Them 2:**  **short-term coping strategies’**  81 Normalisation | Nurse 6: "Let’s say it is work ethics . . . I know this is the job I have to do . . . That’s what keeps me going. After all, I have been trained for this . . . we are on the field in this process . . . who will take care of the patients once we  retreat . . ." p. 164 | U |  |  |
| 82.Refusal to dwell on their experience | Nurse 7: "I tried not to think at first. I think more in the hospital. When I come home, I go to my room and try not to have close contact with family members. I comfort myself saying that these days will pass, only some more days to go, as if it is a temporary period. At first, I was thinking a lot, so my fear, panic and anxiety were very high. Now they decreased, as I am not  thinking about it” p. 64 |  | C |  |
| 83. Avoidance | Nurse 5: ". . . I do not watch any news in the evening, I follow them on the Internet. I muted all of the WhatsApp  groups, I check them out for | U |  |  |

|  | about 5 mins when I am available . . . to see if there is anything involving me . . . I protect myself like this . . ." p.  164 |  |  |  |
| --- | --- | --- | --- | --- |
| 84. Expression of feelings | Nurse 2: ". . . I am not someone who cries a lot but I  am crying" p.164 |  | C |  |
| 85. Distraction | Nurse 3: " I’ve been cooking  more, making up new recipes" p.164 |  | C |  |
| **Them3:**  **Needs**  86 Psychosocial support | Nurse 9: " . . We don’t know coping strategies . . . I feel like consulting an expert, so it would be much much better if psychosocial support were to be provided by psychologists, therapists in related fields by making appointments . . . We really need some sort of support, because we are  under a lot of risk "p. 164 | U |  |  |
| 87. Resource management | Nurse 8: "There are still not enough nurses. Because lack of staff who knows intensive care is felt too much. New appointments have been made, but they are also very recent graduates. The number  of nurses is low" p. 164 | U |  |  |

Kheirandish, E., Rahnama, M., Abdollahimohammad, A., Noorisanchooli, H., and Hashemi, Z. (2020) COVID-19 nurses’ experiences of caring: a qualitative study. *Medical Studies/Studia Medyczne*, *36*(4), 239-245.

| **Finding** | **Illustration from publication (page**  **number)** | **Evidence** | | |
| --- | --- | --- | --- | --- |
| **Them1:**  **Multiple aspects of caring for COVID-19 patients**  88.Physical aspect of caring for COVID-19 patients | "COVID-19 patients are af- fected by severe shortness of breath and quick oxygen saturation decline. Their symptoms changed sudden- ly and their condition changed into coma" (A 42-year-old female nurse with a 16-year working background  stated) p. 242. | Unequivocal (U) | Credible (C)  C | Not Supported (NS) |
| 89. Mental aspects of caring for COVID-19 patients | "The patients think they will die if they do not re- ceive oxygen. Once, I removed a patient’s mask and I said: note that you are OK without oxygen; but the patient said: don’t remove my mask, I will die without oxygen" (a 44-year-old female nurse with an 18-year working  background) p. 242 |  | C |  |
| 90. Emotional aspects of caring for COVID-19 patients | "Although I was scared of COVID-19 patients, I went to them and talked to them. I encouraged them not to be worried and assured them that they would re- cover" (A 41-year-old nurse with 17 years of working background) p. 242. | U |  |  |
| **Them 2:** | "This disease has doubled the  difficulties of our job. We have to | U |  |  |

| **Challenges and requisites of COVID- 19 care**  91. Caring challenges | take the responsibility of caring for COVID-19 patients for a 20-day period. We have been faced with different problems such as wearing the protective clothes, safety glasses, hats, and masks" (A 30- year-old male nurse with 7 years of  working background) p. 243 |  |  |  |
| --- | --- | --- | --- | --- |
| 92.Caring requisites | "Since COVID-19 patients are alone and they do not have any companion, nurses should com- municate with them more than other patients" ( A 30-year- old married male nurse with 8 years of working  back- ground) p. 243 | U |  |  |
| **Them 3:**  **Fear of being infected by the virus**  **and the nurses’ protective self-care**  93.Fear of the disease | "In the early days of work- ing in this ward, I was scared of COVID-19 patients" (A 51-year-old male nurse with 25 years of working background) p. 243. | U |  |  |
| 94. Protective self-care in nurses | "Due to the fear of the disease, we always wear protec- tive clothes when going to the patients" (A 41- year-old female  nurse with 17 years of working  background) p. 243 |  | C |  |

Al Knawy, B., Al-Kadri, H., Elbarbary, M., Arabi, Y., Balkhy, H., and Clark, A. (2019) Perceptions of postoutbreak management by management and healthcare workers of a Middle East respiratory syndrome outbreak in a tertiary care hospital: A qualitative study. *BMJ Open, 9*(5), 017476 [online]. Available at: doi:10.1136/bmjopen-2017-017476 [Accessed: 15 Jun, 2022].

| **Finding** | **Illustration from publication**  **(page number)** | **Evidence** | | |
| --- | --- | --- | --- | --- |
| **Them 1:**  **Factors perceived to contribute to outbreak occurrence**  95.Inadequate implementation of IPC guidelines | “It’s not something you want to see… And when you know, it’s because someone didn’t wash their hands. That’s the bit that killed me. Something simple, it wasn’t that we didn’t have a Da Vinci (Robotic surgery) in the OR. It’s because they didn’t  wash their hands." p. 5 | Unequivocal (U)  **U** | Credible (C) | Not Supported (NS) |
| **Them2:**  **Factors perceived to contribute to success of outbreak control**  96. collective leadership practice | "When we start to see the CEO doing round in the ED, almost every day….and so we can contact him. So you can talk with him. So you can address the problem directly. So he is doing his rounds almost on a daily basis…And he meets with us or the heads of the units and ask, ‘so, how can I help you in this unit?" p. 5  " There was leadership…the commitment came out. That is the first time that we saw each  other twice daily. We were | U |  |  |

|  | spending time together more than we spend with our families. So every day, I will see Doctor  [Leader], every day I will see Doctor [Leader]. So we were always there… So there was – there was a lot of collegiality that – that was really built. Ah, storming led to performing. Meaning that: okay, yes, we had differences of opinions, but at the end of the day, we had to come up with  decisions. … " p. 5. |  |  |  |
| --- | --- | --- | --- | --- |
| 97. Improved mutual trust between front liners and top management | "There seemed to be…probably more teamwork. And there seemed to be a lot more admiration for each other. Yeah, and I think – and that – and it was expressed to me by Doctor  [Leader] 1 day, and I remember the conversation. He said, ‘you know, I can’t believe that these guys kept turning up.’ You know? [Interviewer: Uhum] And ah, and I – I think we grew a bit of mutual respect for each other, out of the way we handled  that, to be honest" p. 5. | U |  |  |
| **Them 4:**  **Factors inhibiting outbreak control** | "I was kept very much in the dark as to what was going on…it wasn’t transcribed. We were –  we were not – we were | U |  |  |

| 98. Poor staff orientation and management ambiguity | only…only fed information that we needed to know. So, rather than team debriefing every day, which I would have appreciated, that didn’t (happen)…So poor communication. And it – it – almost a hierarchy of who needs to know what. And yet, we were in the frontlin "p. 6.  "Well, there was lots of grey… grey areas….Ah, would you put a mask ah, throughout the stay, in the aisle – hospital, ah, in the corridors? Would you not do as some people ah, did? … some people did not ah, do that. There were lots of mixed messages coming from even IPC department. …… Ah, which confused us, which… ah, and so there was lots of variations…some people were more conservative. Some people more – were more liberal in doing these things " p. 6. |  |  |  |
| --- | --- | --- | --- | --- |

| **Them 5:**  **Long-term institutional gains in response to the outbreak management**  99. Tangible improvement in ICP practices | "We – we had a lot of support from Infection Control department…lots of information available. Um, the Infection Control practitioners were on the units day and night, supporting the staff. Management as well….in terms of supplies, equipment, all of that, we didn’t have any issues. It was supplied  – readily available" p. 6. | U |  |  |
| --- | --- | --- | --- | --- |
| 100. Impetus for change in personnel and processes (transformation) | " But we – just we completely redesigned – not only um, it was complete system – restructure and system redesign ….Um, people were practicing in a way that they’ve never done before-  nurses and doctors "p. 6 | U |  |  |

Almutairi, A., Adlan, A., Balkhy, H., Abbas, O., and Clark, A. (2018) “It feels like I'm the dirtiest person in the world.”: Exploring the experiences of healthcare providers who survived MERS-CoV in Saudi Arabia. *Journal of Infection and Public Health, 11*(2), 187-191.

| **Finding** | **Illustration from publication**  **(page number)** | **Evidence** | | |
| --- | --- | --- | --- | --- |
| **Theme 1:**  **caring for others in the defining moments**  101. They believed they caught the virus as  a result of prioritising patient care over their own health | "In the ER . . . we had patients in the hallway. . . and we had patients positive with CORONA behind curtains. . . and I was examining and seeing every single [patient with] CORONA in this hospital by myself and  that’s the price I paid. . .” “ so  the one [who] got positive virus I think was me [the survivor], I think I was too close to the patient because I examine  the patient more than the junior  "pp.188-189 | Unequivocal (U)  U | Credible (C) | Not Supported (NS) |
| 102.Considering their survival as a reward from God for the hard work they were doing for  patients | "Let me tell you something, sometimes I feel that I survived this disease because God knows that I was doing the right  thing. ." p. 189. | U |  |  |
| 103.An ethical or employment related obligation to provide care | "I was afraid, you know when I have patients that has fever I already get paranoid, so when I heard about the outbreak already I wanna go home, but  there’s nothing I can do I have a | U |  |  |

|  | contract, and then I forced myself to stay "p.189 |  |  |  |
| --- | --- | --- | --- | --- |
| **Them 2:**  **prejudice and stigma**  104. Rejection and avoidance by their colleagues and neighbours even after recovery | "I was standing by my window. Here, next to my house, there is a park, and a lot of people here walk to the bus stop, and in the park, there is a distance like maybe from here to you. I was standing here, looking to my window through the curtain, and one nurse who I talk to a lot called, and I was walking here, and I could see her but and I went back I didn’t want her to see me. And she told, “Yeah, I’m at the hospital, and when I have the time I will come to see you.”. . .I said, “Okay, don’t worry, yeah, I’m sorry.” It’s basically very clearly implying that I don’t want to be near you"  p. 189. | U |  |  |
| 105.Ignoring and excluding survivors from their social life or signalling them for special questions | "I felt bad. . .it feels like I’m the dirtiest person in the world; that’s why they have to avoid me. I can’t approach them because they are terrified, you know. I felt bad . . ." p189 | U |  |  |

|  | "A lot of people knew about it. If I go near them, they will ask, “Are you negative? Were you the one who had Corona?” And then they will cover their nose.  Yeah" p.189 |  |  |  |
| --- | --- | --- | --- | --- |
| 106.Peer support without any intimidation behaviours or rejection | "I was born Christian. . .I think the Islamic teaching of just brotherly love and compassion manifested in the way I was treated by A, B, and C, without question you know, and that was the first time I cried, and not because I was in pain but because of the showing of caring. It touched me so much. . .I didn’t cry because I  was in pain, no, but because of the genuine. . .I mean some people who fake it. It was  genuine; it was real. . ." p.189 | U |  |  |
| **Theme 3:**  moments of traumatic fear and despair  107.Feeling hopeless and helpless and frightened of the scenarios that could happen to them. | "In the morning, they said I was positive of Corona, so my feeling that time was very terrible, and of course when I say Corona, all our patients died from Corona" p. 189 | U |  |  |

| 108.Their fear of being infected compounded by adding additional stress. | "So, even I have this kind of thinking, “Oh my God, after few days I will die, after few days I will get intubated, or something like this like that.”. . .I was really scared if I will survive or if I will  be gone that time. . ." p. 189 | U |  |  |
| --- | --- | --- | --- | --- |
| 109.Suffering from the intensity of their experiences after recovery. | "I’m trying to avoid thinking about the incident. . .I still have dreams. Especially yesterday I dreamt about being in ICU seeing everything inserting all the tubes and needles. I saw, I think it will be a big part of my  life already" p. 189 | U |  |  |
| **Theme 4:**  denial and underestimation of the seriousness of the disease  110. At the personal level:  they had different perceptions of the aggressiveness of the infection | “The thing that comes to mind is that life was going on as usual; you know that we had the outbreak in March, so we thought we were over it and Mafee Mushkelah [Arabic for ‘no problem’], and everything in life was going on" p.190. | U |  |  |

| At the official level:  111. official responses to dealing with the situation and containing the virus could not cope with the nature of the infection. | "Create a policy where you alert the staff as soon as you have one case or two cases in the ER; you [i.e. the decision makers] should have alerted all the staff" p.190. | U |  |  |
| --- | --- | --- | --- | --- |

# Appendix 9: JBI Grades of Recommendation ^28^


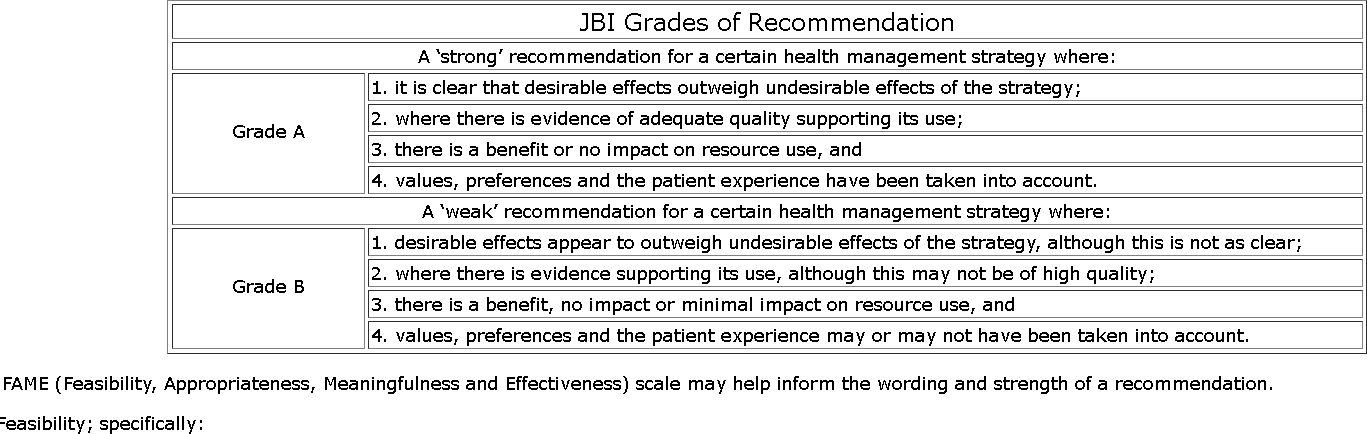


**Reference list**

1. Aromataris, E. and Munn, Z. JBI Manual for Evidence Synthesis. JBI. 2020. https://doi.org/10.46658/JBIMES-20-01. Accessed 10 July 2022.
2. Hannes K, Lockwood C, Pearson A. A Comparative Analysis of Three Online Appraisal Instruments’ Ability to Assess Validity in Qualitative Research. Qual Health Res. 2010;20(12):1736–43.
3. Sarikaya O, Erbaydar T. Avian influenza outbreak in Turkey through health personnel's views: a qualitative study. BMC Public Health. 2007 Dec;7(1):1.
4. Alnazly E, Khraisat OM, Al-Bashaireh AM, Bryant CL. Anxiety, depression, stress, fear and social support during COVID-19 pandemic among Jordanian healthcare workers. PloS one. 2021;16(3):0247679.
5. Kim SC, Sloan C, Chechel L, Redila M, Ferguson J. Severe Burnout and Poor Mental Health Among Healthcare Workers 6 Months After COVID-19 Pandemic Declaration: What Can We Learn for Future Emergencies?. J Nurs Adm. 2021;51(11):554–60. https://nusearch.nottingham.ac.uk/permalink/f/t1e4u0/TN_cdi_proquest_miscellaneous_2585912053. Accessed 10 July 2022.
6. Pourvakhshoori N, Norouzi K, Ahmadi F, Hosseini M, Khankeh H. Nurse in limbo: A qualitative study of nursing in disasters in Iranian context. PloS one. 2017;12(7):0181314.
7. El‐Monshed AH, Amr M, Ali AS, Elmasry YM, Zoromba M. Nurses' knowledge, concerns, perceived impact and preparedness toward COVID‐19 pandemic: A cross‐sectional survey. Int J Nurs Pract. 2021;27(6):13017.
8. Danielis M, Peressoni L, Piani T, Colaetta T, Mesaglio M, Mattiussi E, et al. Nurses’ experiences of being recruited and transferred to a new sub‐intensive care unit devoted to COVID‐19 patients. J Nurs Manag. 2021;29(5):1149–58.
9. Kang HS, Son YD, Chae S, Corte C. Working experiences of nurses during the Middle East respiratory syndrome outbreak. Int J Nurs Pract. 2018;24(5):12664.
10. Khalid I, Khalid TJ, Qabajah MR, Barnard AG, Qushmaq IA. Healthcare workers emotions, perceived stressors and coping strategies during a MERS-CoV outbreak. Clin Med Res. 2016;14(1):7–14.
11. Baker OG, Alamri AA, Aboshaiqah AE. A descriptive study to analyse the disaster preparedness among Saudi nurses through self‐regulation survey. J Nurs Manag. 2019;27(7):1479–84.
12. Bahrami M, Aliakbari F, Aein F. Iranian nurses' perception of essential competences in disaster response: A qualitative study. J Educ Health Promot. 2014; 3:81. https://nusearch.nottingham.ac.uk/permalink/f/t1e4u0/TN_cdi_pubmedcentral_primary_oai_pubmedcentral_nih_gov_4165091. Accessed 10 July 2022.
13. Alsubaie S, Hani Temsah M, Al-Eyadhy AA, Gossady I, Hasan GM, Al-Rabiaah A, et al. Middle East Respiratory Syndrome Coronavirus epidemic impact on healthcare workers' risk perceptions, work and personal lives. J Infect Dev Ctries. 2019;13(10):920–6.
14. Bukhari EE, Temsah MH, Aleyadhy AA, Alrabiaa AA, Alhboob AA, Jamal AA, et al. Middle east respiratory syndrome coronavirus (MERS-CoV) outbreak perceptions of risk and stress evaluation in nurses. J Infect Dev Ctries. 2016;10(8):845–50.
15. Karabulak H, Kaya F. The Relationship Between Psychological Resilience and Stress Perception in Nurses in Turkey During the COVID-19 Pandemic. Nurs Res.2021;29(6):175.
16. Shahrour G, Dardas LA. Acute stress disorder, coping self‐efficacy and subsequent psychological distress among nurses amid COVID‐19. J Nurs Manag. 2020;28(7):1686–95.
17. Al Knawy BA, Al-Kadri HMF, Elbarbary M, Arabi Y, Balkhy HH, Clark A. Perceptions of postoutbreak management by management and healthcare workers of a Middle East respiratory syndrome outbreak in a tertiary care hospital: a qualitative study. BMJ open. 2019;9(5): 017476. doi:10.1136/bmjopen-2017-017476. Accessed 15 Jun 2022.
18. Almutairi AF, Adlan AA, Balkhy HH, Abbas OA, Clark AM. “It feels like I'm the dirtiest person in the world.”: Exploring the experiences of healthcare providers who survived MERS-CoV in Saudi Arabia. J. Infect Public Health. 2018;11(2):187–91.
19. Chegini Z, Arab‐Zozani M, Rajabi MR, Kakemam E. Experiences of critical care nurses fighting against COVID‐19: A qualitative phenomenological study. Nurs Forum. 2021;56(3):571–8.
20. Galehdar N, Kamran A, Toulabi T, Heydari H. Exploring nurses' experiences of psychological distress during care of patients with COVID-19: A qualitative study. BMC psychiatry. 2020;20(1):489–9.
21. Kackin O, Ciydem E, Aci OS, Kutlu FY. Experiences and psychosocial problems of nurses caring for patients diagnosed with COVID-19 in Turkey: A qualitative study. Int J Soc Psychiatry. 2021;67(2):158–67.
22. Kalateh Sadati A, Zarei L, Shahabi S, Heydari ST, Taheri V, Jiriaei R, et al. Nursing experiences of COVID‐19 outbreak in Iran: A qualitative study. Nurs Open. 2021;8(1):72–9.
23. Kandemir D, Yılmaz A, Sönmez B. Professional and psychological perceptions of emergency nurses during the COVID‐19 pandemic: A qualitative study. JJNS. 2022;19(3):12470.
24. Kheirandish E, Rahnama M, Abdollahimohammad A, Noorisanchooli H, Hashemi Z. COVID-19 nurses’ experiences of caring: a qualitative study. Medical Studies/Studia Medyczne. 2020;36(4):239-45.
25. Moradi Y, Baghaei R, Hosseingholipour K, Mollazadeh F. Challenges experienced by ICU nurses throughout the provision of care for COVID‐19 patients: A qualitative study. J Nurs Manag. 2021;29(5):1159–68.
26. Villar RC, Nashwan AJ, Mathew RG, Mohamed AS, Munirathinam S, Abujaber AA, et al. The lived experiences of frontline nurses during the coronavirus disease 2019 (COVID‐19) pandemic in Qatar: A qualitative study. Nurs Open. 2021;8(6):3516–26.
27. Yıldırım N, Aydoğan A, Bulut M. A qualitative study on the experiences of the first nurses assigned to COVID‐19 units in Turkey. J Nurs Manag. 2021;29(6):1366–74.
28. The Joanna Briggs Institute (JBI). Supporting document for the Joanna Briggs Institute Levels of Evidence and Grades of Recommendation. JBI. 2014. https://tinyurl.com/ymsada4w. Accessed15 August 2022.

**File name:** Additional file 1.

**File format:** word document

**Title of data and description of data**:

Appendix 1: Search strategy and the search keywords

Appendix 2: JBI tool to perform critical appraisal for qualitative study

Appendix 3: JBI-QARI data extraction tool for qualitative research

Appendix 4: The JBI Approach to qualitative synthesis (Meta aggregation)

Appendix 5: ConQual summary

Appendix 6: Excluded studies after reading full text

Appendix 7: Characteristics of included studies

Appendix 8: The findings extracted from the included studies

Appendix 9: JBI Grades of Recommendation
